# Spatiotemporal Characterization of Human Early Intervertebral Disc Formation at Single‐Cell Resolution

**DOI:** 10.1002/advs.202206296

**Published:** 2023-03-25

**Authors:** Taifeng Zhou, Yu Chen, Zhiheng Liao, Long Zhang, Deying Su, Zhuling Li, Xiaoming Yang, Xiaona Ke, Hengyu Liu, Yuyu Chen, Ricong Weng, Huimin Shen, Caixia Xu, Yong Wan, Ren Xu, Peiqiang Su

**Affiliations:** ^1^ Department of Spine Surgery Guangdong Provincial Key Laboratory of Orthopedics and Traumatology The First Affiliated Hospital of Sun Yat‐sen University Guangzhou 510080 China; ^2^ State Key Laboratory of Cellular Stress Biology Fujian Provincial Key Laboratory of Organ and Tissue Regeneration School of Medicine Faculty of Medicine and Life Sciences Xiamen University Xiamen 361102 China; ^3^ Guangdong Provincial Key Laboratory of Proteomics and State Key Laboratory of Organ Failure Research School of Basic Medical Sciences Southern Medical University Guangzhou 510515 China; ^4^ Department of Orthopedics Renmin Hospital of Wuhan University Wuhan 430060 China; ^5^ Department of Gynecology and Obstetrics The First Affiliated Hospital of Sun Yat‐sen University Guangzhou 510080 China; ^6^ Research Center for Translational Medicine The First Affiliated Hospital of Sun Yat‐sen University Guangzhou 510080 China

**Keywords:** annulus fibrosus, intervertebral disc formation, notochord, nucleus pulposus, single‐cell RNA sequencing

## Abstract

The intervertebral disc (IVD) acts as a fibrocartilaginous joint to anchor adjacent vertebrae. Although several studies have demonstrated the cellular heterogeneity of adult mature IVDs, a single‐cell transcriptomic atlas mapping early IVD formation is still lacking. Here, the authors generate a spatiotemporal and single cell‐based transcriptomic atlas of human IVD formation at the embryonic stage and a comparative mouse transcript landscape. They identify two novel human notochord (NC)/nucleus pulposus (NP) clusters, SRY‐box transcription factor 10 (SOX10)^+^ and cathepsin K (CTSK)^+^, that are distributed in the early and late stages of IVD formation and they are validated by lineage tracing experiments in mice. Matrisome NC/NP clusters, T‐box transcription factor T (TBXT)^+^ and CTSK^+^, are responsible for the extracellular matrix homeostasis. The IVD atlas suggests that a subcluster of the vertebral chondrocyte subcluster might give rise to an inner annulus fibrosus of chondrogenic origin, while the fibroblastic outer annulus fibrosus preferentially expresseds transgelin and fibromodulin . Through analyzing intercellular crosstalk, the authors further find that notochordal secreted phosphoprotein 1 (SPP1) is a novel cue in the IVD microenvironment, and it is associated with IVD development and degeneration. In conclusion, the single‐cell transcriptomic atlas will be leveraged to develop preventative and regenerative strategies for IVD degeneration.

## Graphical abstract

Notochord‐to‐nucleus pulposus transition is the key feature of early intervertebral disc (IVD) formation. This study provides a single‐cell view of early IVD formation in both humans and mice, and the transcriptomic features of developing notochord/nucleus pulposus cells, vertebral chondrocytes, and annulus fibrosus cells are characterized. This developmental atlas can be leveraged in the development of regenerative strategies for IVD degeneration.

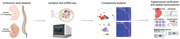

## 1 Introduction

The axial skeleton protects our body during daily physical movement, including the vertebral bodies (VBs), intervertebral discs (IVDs), tendons/ligaments, and muscles. IVDs act as fibrocartilaginous joints to anchor adjacent VBs, which is essential for the mechanical stabilization.^[^ ^1^ ^]^ Mature IVDs consist of the central nucleus pulposus (NP), surrounding annulus fibrosus (AF), and end plate (EP) that adjoins the VB. The NP originates from the embryonic notochord (NC), while the AF and EP are derivates of the mesenchymal sclerotome.^[^ ^2^ ^]^ The characteristic feature of embryonic IVD formation is the NC‐to‐NP transition, after which NC cells are thought to begin reducing and even disappear in postnatal human IVDs.^[^ ^1^, ^3^ ^]^ NC cells have been reported to be the precursors of all NP cells,^[^ ^4^ ^]^ and the loss of NC cells is closely associated with IVD degeneration (IVDD) onset.^[^ ^5^ ^]^

IVDD is known to be a major cause of low back pain, which can be severe and often leads to disability.^[^ ^6^ ^]^ A poor understanding of the cellular and molecular basis underlying IVD formation and degeneration limits the development of disease‐modifying therapeutics for IVDD.^[^ ^1^ ^]^ Recently, single‐cell RNA sequencing (scRNA‐seq) has provided a powerful alternative to study cellular heterogeneity and to help uncover regulatory relationships among genes during organ development and degeneration.^[^ ^7^ ^]^ To develop regenerative strategies for IVDD, some recent scRNA‐seq studies revealed the cellular heterogeneity within adult mature IVDs in both humans and mice.^[^ ^4^, ^8^ ^]^ However, limited information is known about early human IVD formation, especially in regard to the cellular heterogeneity during the embryonic NC‐to‐NP transition, due to the lack of high‐precision and unbiased resolutions for distinguishing cell populations during early human IVD formation.

In this study, we aimed to provide a single‐cell view of human early IVD formation. We profiled 177 725 cells from the axial skeleton across 13 human embryos at different gestational weeks, along with 22 838 cells from the mouse axial skeleton. Combined with lineage tracing and spatial transcriptome analysis, we comprehensively characterized the transcriptome features of developing NC/NP cells, vertebral chondrocytes, and AF cells during early IVD formation. We identified two novel NC/NP clusters as well as potential inner AF/EP progenitors, and we decoded the intercellular crosstalk with respect to early IVD formation. Our results provide new cellular‐level insights into the transcriptional alterations associated with early IVD formation, and this could be leveraged in the development of preventative and regenerative strategies for IVDD.

## 2 Results

### 2.1 Single‐Cell Profiling of the Embryonic Axial Skeleton Cell Atlas during Early IVD Formation

**Figure 1:**
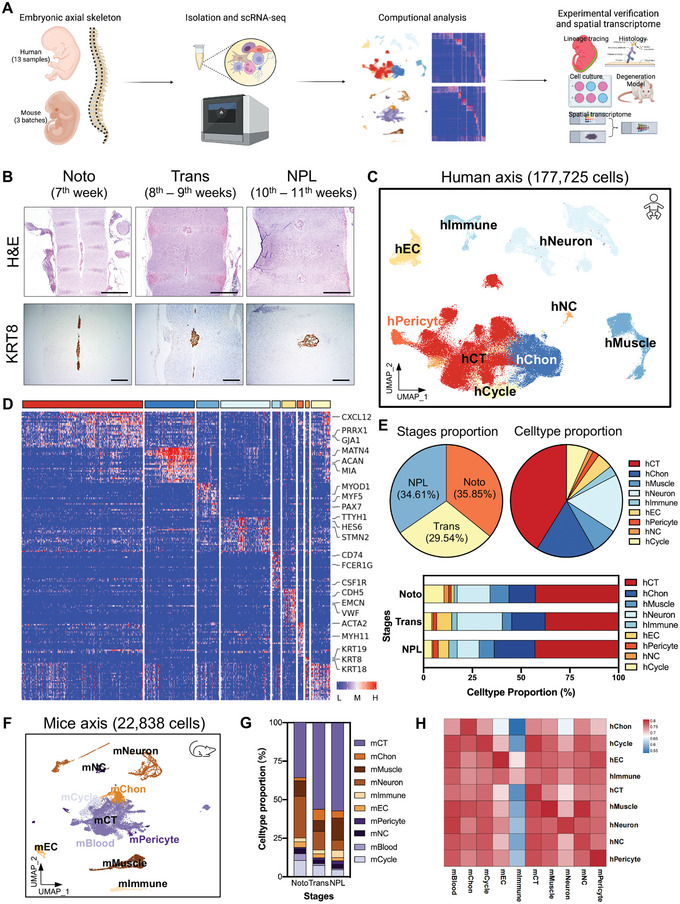
Single‐cell profiling of the embryonic axial skeleton cell atlas during early IVD formation. A) Schematic image of the experimental workflow. B) Representative images of human embryonic axial skeleton sections at different developmental stages. Upper lane, representative images of H&E staining; lower lane, representative images of KRT8 IHC staining. Scale bar, 500 µm. C) UMAP visualization of human embryonic axial skeleton cells identified nine different clusters. Each dot corresponds to one single cell colored according to cell cluster. D) Heatmap showing the scaled expression of differentially expressed genes for each human cluster. E) Proportion of developmental stages and cell clusters (upper lane) and fraction of human cell clusters at the Noto, Trans, and NPL stages (lower lane). F) UMAP visualization of mouse embryonic axial skeleton cells identified 10 different clusters. Each dot corresponds to one single cell colored according to cell cluster. G) Fraction of mouse cell clusters at the Noto, Trans, and NPL stages. H) Heatmap showing pairwise Pearson correlations in the global transcriptome between human and mouse cell clusters.

Human fetuses were obtained from the First Affiliated Hospital of Sun Yat‐sen University from donors who provided informed consent for the use in research of fetal material arising from the termination of their pregnancy. Morphologically normal embryos at the 7th to 11th weeks of gestation, when the NP undergoes a chronological transition from a rod‐like notochord to segmented fibrocartilaginous tissue,^[^ ^9^ ^]^ were staged by an ultrasound system and collected. We microdissected and dissociated the axial skeleton, including the presumptive VB, annulus fibrosus (AF), NP, and other adherent soft tissues (i.e., muscles, tendons, and ligaments), for scRNA‐seq. A total of 13 fetuses were analyzed using chromium (10x Genomics, Pleasanton, CA, USA) (**Figure** **1**A). Based on the morphology of the developing IVDs, these specimens were grouped into three stages: the notochord stage (Noto, week 7, *n* = 4), the transition stage (Trans, weeks 8 and 9, *n* = 4), and the nucleus pulposus‐like stage (NPL, weeks 10 and 11, *n* = 5) (Figure 1B).

A dataset of 177 725 cells was generated after quality control and doublet exclusion filtering to remove cells with abnormal gene detection (<500 genes or >6000 genes) and high mitochondrial gene content (>15%) (Figure 1C and Figure S1A–E, Supporting Information). We defined the following nine distinct clusters according to the expressions of known markers (Figure 1C and Figure S2A, Supporting Information): the Cartilage (hChon, clusters 0–5; 30 376 cells), the Connective Tissue (hCT, clusters 6–19; 73203 cells), the Notochord (hNC, clusters 20 and 21; 2336 cells), the Muscle (hMuscle, clusters 22–24; 13 489 cells), the Neuron (hNeuron, clusters 25–40; 29 833 cells), the Immune (hImmune, clusters 41–44; 5064 cells), the Endothelium (hEC, clusters 45–47; 8330 cells), the Pericyte (hPericyte, cluster 48; 3368 cells), and the Cell in Cell Cycle (hCycle, clusters 49 and 50; 11 726 cells) clusters. Based on differential expression gene analysis, we listed the most significantly enriched genes defining each lineage (Figure 1D and Table S1, Supporting Information). hChon was distinguished by the expressions of *ACAN*, *MIA*, *SOX9*, *COL2A1*, *MATN4*, and *HAPLN1* ^[^ ^10^ ^]^ (Figure 1D and Figure S3A, Supporting Information). hCT was characterized by the expressions of *CXCL12*, *PRRX1*, and *GJA1*,^[^ ^11^ ^]^ as well as other markers such as *FOXC2*, *TWIST1*, and *EGFL6* ^[^ ^12^ ^]^ (Figure 1D and Figure S3A, Supporting Information). hNC was identified by the expressions of *KRT18*, *KRT19*, *KRT8*, *CD24*, and *SHH* ^[^ ^9^, ^13^ ^]^ (Figure 1D and Figure S3A, Supporting Information). hMuscle was marked by the expressions of *MYOD1*, *MYF5*, and *PAX7*.^[^ ^14^ ^]^ hNeuron was marked by the expressions of *TTYH1*, *HES6*, and *STMN2*.^[^ ^15^ ^]^ hImmune was marked by the expressions of *CD74*, *FCER1G*, and *CSF1R*.^[^ ^16^ ^]^ hEC was marked by the expressions of *CDH5*, *EMCN*, and *VWF*.^[^ ^4^, ^17^ ^]^ hPericyte was marked by the expressions of *ACTA2* and *MYH11*.^[^ ^18^ ^]^ Finally, hCycle was marked by the expressions of *TOP2A*, *HIST1H4C*, and *HIST1H1A* ^[^ ^19^ ^]^ (Figure 1D and Table S1, Supporting Information). The proportions of hNC at the three stages (2.50%, 0.74%, and 0.58%, respectively) as well as those of hCycle (10.57%, 4.29%, and 4.46%, respectively) dramatically decreased with IVD development. In contrast, the proportions of hEC (1.49%, 7.72%, and 5.40%, respectively) and hPericyte (1.23%, 1.83%, and 2.64%, respectively) were elevated (Figure 1E and Figure S2B, Supporting Information). These results reflected increased angiogenesis, which is essential for endochondral ossification during VB formation.

To understand the similarities and differences between humans and mice during early IVD formation, we dissected the mouse axial skeleton at embryonic days 11.5, 13.5, and 15.5 (E11.5, Noto; E13.5, Trans; E15.5, NPL), followed by scRNA‐seq using 10x Genomics chromium (Figure 1A). A dataset of 22 838 mouse cells was generated after quality control (Figure 1F and Figure S1F–H, Supporting Information). Similarly, 10 distinct lineages were identified based on the expressions of known markers (Figure 1F,G and Figure S3B, Supporting Information), as follows: the Cartilage (mChon, cluster 0; 1136 cells), the Connective Tissue (mCT, clusters 1–15; 11582 cells), the Notochord (mNC, clusters 16–18; 589 cells), the Muscle (mMuscle, clusters 19–22; 2361 cells), the Neuron (mNeuron, clusters 23–28; 3264 cells), the Immune (mImmune, clusters 29 and 30; 746 cells), the Endothelium (mEC, cluster 31; 630 cells), the Pericyte (mPericyte, cluster 32; 382 cells), the Cell in Cell Cycle (mCycle, clusters 33 and 34; 1706 cells), and the Blood Cell (mBlood, cluster 35; 442 cells) lineages, respectively. Similarly, mChon was marked by the expressions of *Col2a1*, *Matn4*, *Hapln1*, and *Mia*; mCT was identified by the expressions of *Igf1*,^[^ ^20^ ^]^ *Egfl6*, *Prrx1*, and *Cxcl12*; mNC highly expressed *Krt8*, *Krt18*, and *Krt19*; mMuscle was marked by the expressions of *Myog*,^[^ ^21^ ^]^ *Myod1* and *Actc1*;^[^ ^22^ ^]^ mNeuron was defined by the expressions of *Stmn2*, *Tubb3*,^[^ ^23^ ^]^ *Nefm*,^[^ ^24^ ^]^ and *Map2*;^[^ ^25^ ^]^ mImmune was marked by the expressions of *Fcer1g*, *Lyz2*,^[^ ^26^ ^]^ and *Csf1r*; mEC was marked by the expressions of *Cdh5*, *Emcn*, and *Pecam1*;^[^ ^27^ ^]^ mPericyte was marked by the expressions of *Acta2* and *Myh11*; mCycle was marked by the expressions of *Hist1h1b*, *Top2a*, and *Mki67*; and mBlood was marked by the expression of *Hbb‐y* ^[^ ^28^ ^]^ (Figure S3B and Table S2, Supporting Information). Finally, the comparison of transcriptomic differences in axial clusters revealed a high degree of cluster similarity between humans and mice (Figure 1H and Figure S4, Supporting Information).

### 2.2 Temporal Characterization of Developing NC/NP during Early IVD Formation

**Figure 2:**
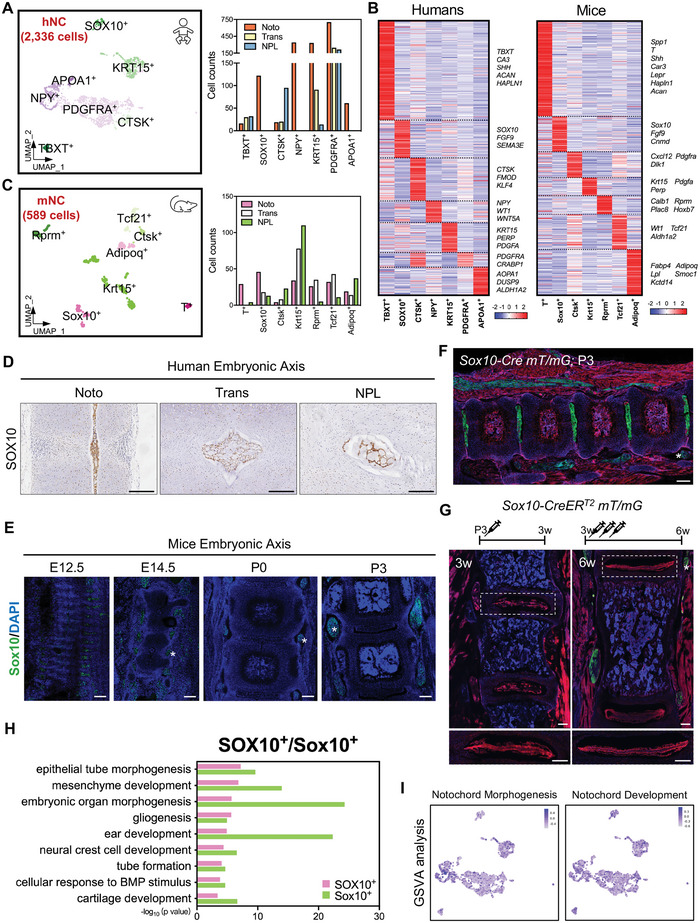
Characterization of developing NC/NP cells during early IVD formation. A) UMAP visualization (left panel) of the seven subclusters of 2336 NC/NP cells defined during human early IVD formation and cell counts (right panel) of each human NC/NP cell subcluster at the Noto, Trans, and NPL stages. B) Heatmap revealing the scaled expression of differentially expressed genes for each human (left) and mouse (right) NC/NP cell subcluster. C) UMAP visualization (left panel) of the seven subclusters of 589 NC/NP cells defined during mouse early IVD formation and cell counts (right panel) of each mouse NC/NP cell subcluster at the Noto, Trans, and NPL stages. D) Representative IHC images of SOX10 in human axial skeleton sections at the indicated developmental stages. Scale bar, 200 µm. E) Representative IF images of Sox10 in mouse axial skeleton sections at indicated developmental stages. Asterisk indicates DRG. Scale bar, 200 µm. F) Representative images of lineage tracing in *Sox10‐Cre;mT/mG* postnatal mouse IVDs. Scale bar, 200 µm. Asterisk indicates DRG. G) Representative images of lineage tracing in *Sox10‐CreERT2;mT/mG* mouse IVDs. Schematic images of tamoxifen administration are indicated on the top of each image. Asterisk indicates DRG. Scale bar, 200 µm. H) Representation analysis of GO categories showing different functions for the SOX10^+^ and Sox10^+^ clusters. I) UMAP plot color‐coded for pathway activities scored by GSVA per cell within hNC subpopulations. Notochord morphogenesis (GO:0048570): the process in which the anatomical structures of the notochord are generated and organized. Notochord development (GO:0030903): the process whose specific outcome is the progression of the notochord over time, from its formation to the mature structure.

We identified hNC according to the expressions of *KRT19*, *KRT18*, and *KRT8* (Figure S5A, Supporting Information), and it appeared to develop into two main subpopulations (Figure 1C and Figures S1C and S5B,C, Supporting Information): cluster 20 (preferentially expressing *BST2*, *PDGFRA*, and *WT1*) and cluster 21 (preferentially expressing *EPCAM*, *CA3*, and *PERP*) (Figure S5D, Supporting Information). To better determine the composition of hNC, we further divided hNC into the following seven subpopulations (**Figure** **2**A, B and Table S3, Supporting Information): TBXT^+^, marked by the expressions of canonical markers, such as *SHH*, *CA3*, and *TBXT*;^[^ ^8a^ ^]^ SOX10^+^, identified by the expressions of *SOX10*, *FGF9*, and *SEMA3E*; CTSK^+^, defined by the expressions of *CTSK*, *FMOD*, and *KLF4*; NPY^+^ (preferentially expressing *NPY*, *WT1*, and *WNT5A*); KRT15^+^ (preferentially expressing *KRT15*, *PERP*, and *PDGFA*); PDGFRA^+^ (preferentially expressing *PDGFRA* and *CRABP1*), and APOA1^+^ (preferentially expressing *APOA1*, *DUSP9*, and *ALDH1A2*). Similarly, the mNCs were also divided into seven subclusters (Figure 2B,C and Table S4, Supporting Information), as follows: T^+^, Sox10^+^, Ctsk^+^, Krt15^+^, Rprm^+^, Tcf21^+^, and Adipoq^+^. Referring to the temporal distribution, SOX10^+^ and Sox10^+^ subpopulations were predominantly at the Noto stage, while CTSK^+^ and Ctsk^+^ clusters increased along with IVD development (Figure 2A,C and Figure S5E–I, Supporting Information). However, the proportions of TBXT^+^ and T^+^ and those of KRT15^+^ and Krt15^+^ showed the opposing tendencies in humans and mice (Figure 2A,C and Figure S5E–I, Supporting Information).

The characteristic genes of SOX10^+^ were enriched in previously reported markers of notochordal precursors (*FGF9* and *FGF20*),^[^ ^29^ ^]^ cell fate determination (*SOX11*), and stem cell maintenance (*HMGB3* and *HMGA1*) (Figure 2B and Table S3, Supporting Information). These molecular signatures were also similarly expressed in Sox10^+^ (Figure 2B and Table S4, Supporting Information). The expressions of both SOX10 in the human fetal spine and Sox10 in the mouse embryonic spine decreased with the NC‐to‐NP transition (Figure 2D,E). Lineage tracing was then performed to better characterize Sox10^+^ subpopulation during early IVD formation. First, we crossed *Sox10‐Cre* mice with *mT/mG* mice, in which transgene expression of tdTomato converts to the expression of enhanced green fluorescent protein (GFP) following Cre‐mediated excision.^[^ ^30^ ^]^ The GFP^+^ Sox10 lineage cells (Sox10^+^ cells and their descendants) included most NP cells and dorsal root ganglion (DRG) cells in the postnatal mouse axis (Figure 2F). Next, we used the CreER^T2^ system to achieve temporal control of Cre recombinase through tamoxifen injection to explore Sox10‐expressing cells after the NC‐to‐NP transition. Tamoxifen administration did not mark any NP cells at either postnatal day 3 (P3) or week 3 (3w) in *Sox10‐CreER^T2^; mT/mG* mice (Figure 2G), which was similar to the outcome of *Shh*‐expressing NC/NP cells.^[^ ^31^ ^]^

The notochordal signaling is responsible for neural tube closure, while its defects result in spina bifida and exencephaly.^[^ ^32^ ^]^ Gene Ontology (GO) term enrichment analysis revealed the capacity of SOX10^+^ and Sox10^+^ subpopulations in regulating epithelial tube morphogenesis, mesenchyme development, tube formation, and cartilage development (Figure 2H). In addition, we found that SOX10^+^ subpopulation highly expressed *BMP7*, *SPINT2*, and *CLDN3/4/6/7* (Table S3, Supporting Information), which are critical for neural tube closure.^[^ ^33^ ^]^ The single‐cell regulatory network inference and clustering (SCENIC)^[^ ^34^ ^]^ showed that SOX10^+^ subpopulation was enriched in regulons including *SOX10*, *FOXG1*, and *SIX1* (Figure S6A, Supporting Information). GO analysis further revealed that the downstream target genes of *SOX10* regulon were enriched in “Notch signaling pathway,” “neural tube closure,” and “positive regulation of activin receptor signaling pathway” (Figure S6B,C and Table S5, Supporting Information). Notch signaling pathway is evolutionarily conserved and essential for midline development,^[^ ^35^ ^]^ and activin signaling is required for notochordal cell differentiation.^[^ ^36^ ^]^ Taken together, our results indicated that *SOX10* is a novel marker for early NC cells in both humans and mice, and that SOX10^+^ cells may be potential notochordal stem/progenitor cells.

The characteristic genes of APOA1^+^ were enriched in retinoic acid binding (*APOA1*),^[^ ^37^ ^]^ retinoic acid synthesis (*ALDH1A2*), and the transportation of retinol (*RBP1*) (Figure S7A and Table S3, Supporting Information). Although retinoic acid is a well‐known teratogen capable of causing neural tube defects, a physiological level of retinoic acid is required for the formation of neural tubes/spinal cords.^[^ ^38^ ^]^ In mNC subpopulations, we found that the Tcf21^+^ subpopulation preferentially expressed *Wt1* and *Wnt5a* (Figure S7B, Supporting Information), which are required for anteroposterior axis specification.^[^ ^39^ ^]^ GO enrichment analysis showed that the characteristic genes of the Tcf21^+^ subpopulation were associated with “anterior/posterior pattern specification,” “regulation of Wnt signaling pathway,” “segmentation,” “neural tube development,” and “neural tube closure” (Figure S7C, Supporting Information). Furthermore, gene set variation analysis (GSVA) showed that the Tcf21^+^ subpopulation was closely associated with “notochord morphogenesis” (Figure S7D, Supporting Information), the process in which the anatomical structures of the notochord are generated and organized (GO:0048570). These indicated that the Tcf21^+^ subpopulation might be involved in regulating notochord development and neural tube closure. Meanwhile, GO enrichment analysis showed that the characteristic genes of the Rprm^+^ subpopulation are associated with “epithelial tube morphogenesis,” “neural tube development,” “neural tube formation,” “neural tube closure,” and “regulation of retinoic acid receptor signaling pathway” (Figure S7C, Supporting Information). This indicated that the Rprm^+^ subpopulation might be involved in retinoic acid signaling pathway and neural tube closure.

The NPY^+^ subpopulation was exclusively distributed at the Noto stage and preferentially expressed *WT1* and *WNT5A* (Figure 2A,B and Table S3, Supporting Information), which are required for anteroposterior axis specification. Accordingly, GSVA showed that the NPY^+^ subpopulation was closely associated with “notochord morphogenesis” (Figure 2I). The pseudotime analysis revealed a developmental trajectory among hNC subclusters (Figure S7E,F, Supporting Information), in which the NPY^+^ subpopulation was located at the start. The route then seemed to develop into two branches (Branch 1 and 2), the CTSK^+^ and the TBXT^+^ subclusters were mainly distributed at each end. These indicated that NPY^+^ subpopulation might be essential for early IVD formation.

Taken together, we revealed the heterogeneous cellular map of NC/NP during early IVD formation in humans and mice, and then we validated *Sox10*‐expressing early NC cells through lineage tracing.

### 2.3 TBXT^+^ and CTSK^+^ Are Two Major Matrisome Clusters That Regulate Extracellular Matrix (ECM) Homeostasis during Early IVD Formation

**Figure 3:**
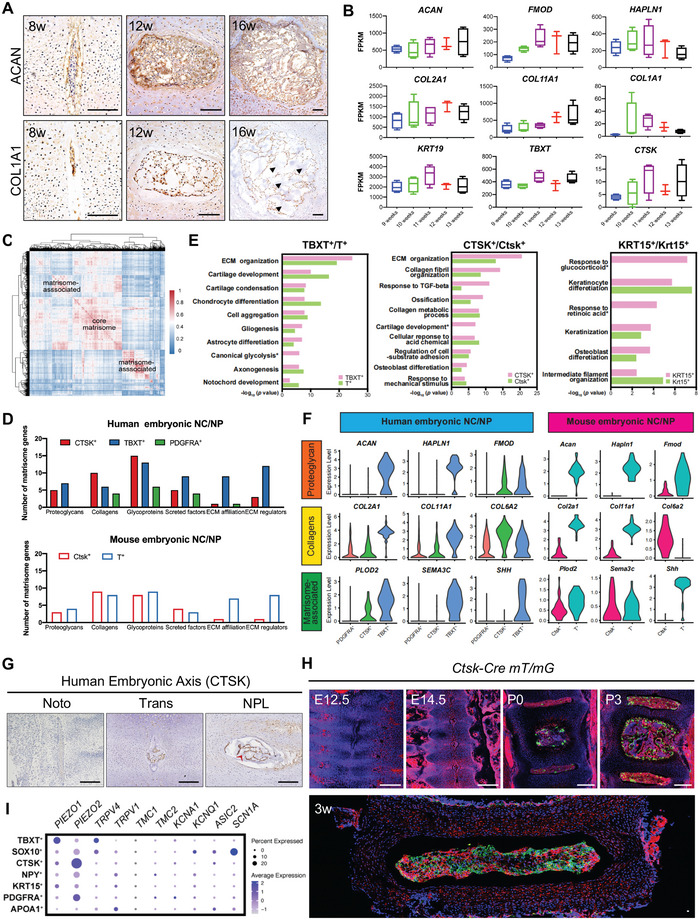
Characterization of matrisome clusters during early IVD formation. A) Representative IHC images of ACAN (upper lane) and COL1A1 (lower lane) in developing human IVDs at the indicated developmental stages. Arrows indicate the increased ECM in the human NP region. Scale bar, 100 µm. B) FPKM expression of the indicated signature genes in developing human NC/NP tissues captured by LCM during early IVD formation. *N* of 9, 10, 11, and 13 weeks = 4; *N* of 12 weeks = 3. C) Heatmap showing pairwise Pearson correlations of expressed matrisome genes in human developing NC/NP cells. D) The number of expressed genes associated with six matrisome patterns in the indicated human (upper) and mouse (lower) NC/NP cell subclusters. E) Representation analysis of GO categories showing different functions for TBXT^+^/T^+^ (left), CTSK^+^/Ctsk^+^ (middle), and KRT15^+^/Krt15^+^ clusters (right). Asterisks indicate the GO categories only enriched in human NC/NP subclusters. F) Violin plots showing the expression levels of representative genes associated with six matrisome patterns in the indicated human (left) and mouse (right) NC/NP cell subclusters. G) Representative IHC images of CTSK in human axial skeleton sections at the indicated developmental stages. The red arrow indicates the ECM in the NP region at the NPL stage. Scale bar, 200 µm. H) Representative images of lineage tracing in the *Ctsk‐Cre;mT/mG* mouse IVD. Scale bar, 200 µm. I) Dot plot showing the mean expression of selected mechanosensitive ion channel genes among seven human NC/NP cell subpopulations. The dot size indicates the percentage of cells in subclusters with detected expression.

Mature NP contains various ECM proteins, including proteoglycans, fibrillar collagens, and elastin fibers, which maintain the height and hydrostatic pressure of IVDs.^[^ ^1^ ^]^ Within mature NP, randomly organized collagen fibers provide tensile strength, and proteoglycans create a large osmotic swelling pressure.^[^ ^40^ ^]^ We found that ECM proteins, such as ACAN and COL1A1, dramatically increased with IVD development (**Figure** **3**A). Our in‐house bulk RNA‐seq results of human fetal NC captured by laser capture microdissection (LCM) also revealed elevated expressions of collagens (*COL2A1* and *COL11A1*) and proteoglycans (*ACAN* and *FMOD*) from nine to 13 weeks of gestational age (Figure 3B and Table S6, Supporting Information).

ECM genes are categorized into core matrisome (collagens, proteoglycans, and glycoproteins) and matrisome‐associated (secreted factors, ECM affiliation, and ECM regulators) groups (matrisomeproject.mit.edu).^[^ ^41^ ^]^ We first evaluated the average expression of six modules in these seven hNC subpopulations (Figure S8A, Supporting Information) and compared the expression abundance of ECM genes in these hNC subpopulations (Figure 3C,D and Figure S8B, Supporting Information). The ECM homeostasis was mainly regulated by both the TBXT^+^ and CTSK^+^ subpopulations, and GO analysis also showed that their characteristic genes were enriched in “ECM organization” (Figure 3E). More specifically, the collagens were preferentially expressed in the CTSK^+^ subpopulation, and GO analysis also showed that the characteristic genes of CTSK^+^ were associated with “collagen fibril organization” and “collagen metabolic organization” (Figure 3E). Thus, CTSK^+^ subpopulation might preferentially contribute to collagens in NP tissue, providing tensile strength. The TBXT^+^ subpopulation preferentially expressed *ACAN* (Figure 3F and Figure S8C, Supporting Information), which encoded the most abundant proteoglycan, aggrecan, within NP. Thus, the TBXT^+^ subpopulation might mainly contribute to the proteoglycans in NP tissue, creating a large osmotic swelling pressure. In humans, PDGFRA^+^ subpopulation was also enriched in “ECM organization” and “collagen fibril organization” (Figure S8D, Supporting Information).

Furthermore, SCENIC regulatory network analysis revealed that the TBXT^+^ subpopulation was enriched for regulons such as *SOX9*, *SOX5*, and *SOX6* (Figure S6A,D, Supporting Information), which are critical regulators of *ACAN* expression.^[^ ^42^ ^]^ Meanwhile, GO analysis revealed that the characteristic genes of the CTSK^+^ subpopulation were associated with “response to TGF‐beta” (Figure 3E), which is essential for NP cell maintenance and ECM homeostasis.^[^ ^43^ ^]^ The major ligand that activates TGF‐*β* pathway in NC/NP cells, *TGFB3*,^[^ ^36^ ^]^ was predominantly expressed in the CTSK^+^ subpopulation and elevated with IVD development (Figure S8C, Supporting Information). It is accepted that the ligand binds to TGFBR2 at the cell surface, which then recruits TGFBR1 to form a heterotetrameric complex with an additional TGFBR1 and TGFBR2.^[^ ^44^ ^]^ We found that *TGFBR2* was preferentially expressed in the CTSK^+^ and the TBXT^+^ subpopulations (Figure S8C, Supporting Information). These indicate that the CTSK^+^ might modulate ECM homeostasis through the TGF‐*β* signaling pathway. Taken together, these bioinformatic clues suggest that the TBXT^+^ subpopulation may mainly contribute to proteoglycan homeostasis under the regulation of *SOX9*, *SOX5*, and *SOX6*, while the CTSK^+^ subpopulation may be mainly involved in collagen homeostasis through the TGF‐*β* signaling pathway.

In the human fetal spine, CTSK was scarcely detected at the Noto stage, while its expression increased with ECM formation in developing NC/NP cells (Figure 3A,G), which was consistent with the elevated expression of *CTSK* in human fetal LCM specimens (Figure 3B). Next, we crossed *Ctsk‐Cre* mice with *mT/mG* mice to further investigate the dynamic expression of Ctsk during early IVD formation (Figure 3H). We found that GFP^+^ Ctsk lineage cells began to appear at P0 in the NP as well as in the ossification center of the VBs, rather than the EP and AF. Of note, the number of GFP^+^ Ctsk lineage cells increased with IVD development, which was consistent with our abovementioned results. This finding indicated that CTSK is a late marker of developing NC/NP cells and that CTSK^+^ might be responsible for ECM homeostasis in fibrocartilaginous NP.

The mature NP in the IVD plays a role in absorbing compressive forces during normal motion and activities. We found that the CTSK^+^ subpopulation was enriched in “response to mechanical stimulus” (Figure 3E). We then compared the expressions of different mechanosensitive ion channels and found that CTSK^+^ cells highly express *PIEZO2*, while TBXT^+^ cells preferentially express *PIEZO1* and *TRPV4* (Figure 3I). In mice, T^+^ preferentially expressed *Trpv4* (Figure S8E, Supporting Information). This indicated that CTSK^+^ and TBXT^+^ subpopulations might be responsible for mechanotransduction within developing NC/NP cells.

In addition to proteoglycans and collagens, cytokeratin, which is an important component of intermediate filaments, was reported to be present in the human notochord.^[^ ^45^ ^]^ We found that KRT15^+^ subpopulation was enriched for cytokeratin (*KRT15, KRT17*, and *KRT19*) and epithelial markers (*EPCAM* and *PERP*) (Tables S3 and S4 and Figure S8F, Supporting Information), and GO analysis showed that KRT15^+^ was enriched in terms associated with “keratinization” and “intermediate filament organization” (Figure 3E). This indicated that KRT15^+^ is responsible for the epithelial characteristics of the notochord.^[^ ^45a^ ^]^

Taken together, we demonstrated the cellular heterogeneity and molecular dynamics related to ECM homeostasis in developing NC/NP cells, and we propose that TBXT^+^ and CTSK^+^ are two major matrisome clusters that act during human early IVD formation.

### 2.4 Temporal and Functional Characterization of Developing Vertebral Chondrocytes

**Figure 4:**
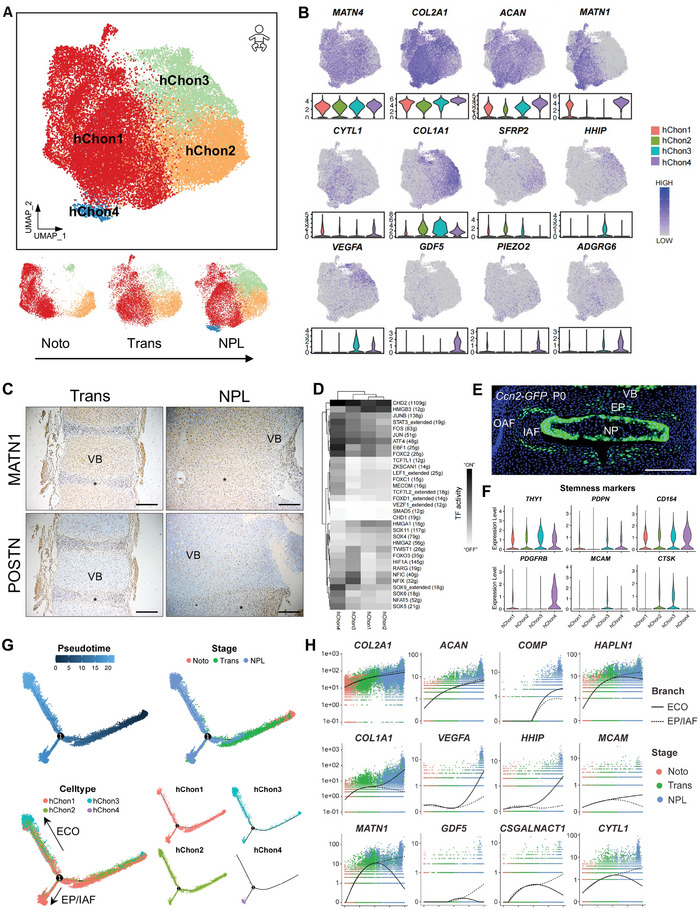
Characterization of vertebral chondrocytes during early IVD formation. A) UMAP visualization (upper) of the four vertebral chondrocyte subclusters defined during human early IVD formation at the Noto, Trans, and NPL stages (lower). B) UMAP plots and violin plots showing the expressions of *MATN4*, *COL2A1*, *ACAN*, *MATN1*, *CYTL1*, *COL1A1*, *SFRP2*, *HHIP*, *VEGFA*, *GDF5*, *PIEZO2*, and *ADGRG6* on UMAP. C) Representative IHC images of MATN1 (upper) and POSTN (lower) in human axial skeleton sections at the indicated developmental stages. Asterisks indicate AF. Scale bar, 200 µm. D) Heatmap revealing binary regulon activities analyzed with SCENIC in each vertebral chondrocyte subpopulation. “ON” indicates active regulons, and “OFF” indicates inactive regulons. E) Representative fluorescent images of the *Ccn2‐GFP* mouse IVD section at P0. OAF, outer annulus fibrosus; IAF, inner annulus fibrosus; VB, vertebral body; EP, end plate; NP, nucleus pulposus. Scale bar, 200 µm. F) Violin plot showing the expression of stem/progenitor markers, including *THY1*, *PDPN*, *CD164*, *PDGFRB*, *MCAM*, and *CTSK*. G) Monocle pseudotime trajectory axis revealing the progression of human vertebral chondrocytes. ECO, endochondral ossification; EP/IAF, end plate/inner annulus fibrosus. H) Expression of selected differentially expressed genes for each branch. The cells were ordered in pseudotime analyzed with Monocle and colored in developmental stages. The full line represents the ECO, while the dotted line represents the EP/IAF. ECO, endochondral ossification; EP/IAF, end plate/inner annulus fibrosus.

To characterize the dynamics and functions of developing vertebral chondrocytes, we aimed to partition hChon into four subclusters (**Figure** **4**A; Figure S9A and Table S7, Supporting Information); however, mChon was ultimately only subdivided into Col2a1^+^ and Col1a1^+^ clusters due to the limited amount of cell number (Figure S9B, Supporting Information). hChon1, which is characterized by the expressions of *MATN1* and *CYTL1*, is the major component of VB anlagen, especially at the Noto stage (Figure 4A,B). The expression of *MATN1* was restricted in epiphyseal chondrocytes (Figure 4C),^[^ ^46^ ^]^ and the characteristic genes of hChon1 were enriched in terms associated with “cartilage development” and “ECM organization” (Figure S9C, Supporting Information). This finding indicated that hChon1 might form central cartilage primordia. hChon2 preferentially expressed *COL1A1*, *POSTN*, and *SFRP2* (Figure 4B and Table S7, Supporting Information), while POSTN (Figure 4C) and SFRP2^[^ ^47^ ^]^ were markers of the perichondrial cells that surrounded and lined chondrogenic condensations. 33 core regulons were filtered to discriminate these hChon clusters by SCENIC analysis (Figure 4D and Table S8, Supporting Information). The axial cartilage at the Noto stage mainly consisted of hChon1 and hChon2, both of which exhibited strong enrichment of *HMGA1*, *HMGB3*, and *SOX11* (Figure 4D). More specifically, SOX11 is widely expressed during early embryogenesis and is essential for stem cell/progenitor survival.^[^ ^48^ ^]^ HMGA1 binds directly to the *SOX9* promoter to induce its expression,^[^ ^49^ ^]^ and the expression of SOX9 is essential for chondrogenic mesenchymal condensation.^[^ ^50^ ^]^

hChon3 increased drastically with VB development (Figure 4A) and highly expressed *VEGFA* (angiogenesis) and *HHIP* (IHH target gene) (Figure 4B). The characteristic genes of hChon3 were enriched in “response to hypoxia,” “ossification,” and “osteoblast differentiation” (Figure S9C, Supporting Information). Moreover, the *HIF1A*, *RARG*, and *NFIX* regulons were specific to hChon3 (Figure 4D). HIF1A is a master regulator of hypoxic vascular responses,^[^ ^51^ ^]^ and RARG is required for the differentiation of hypertrophic chondrocytes;^[^ ^52^ ^]^ both are necessary for endochondral ossification. These results indicated a potential role of hChon3 in regulating endochondral ossification.

hChon4 was exclusively distributed at the NPL stage (Figure 4A), and was marked by the expressions of *GDF5*, *PIEZO2*, and *ADGRG6* (Figure 4B). GDF5 is required for normal joint development in both humans and mice,^[^ ^53^ ^]^ and *Gdf5^−/−^* mice showed smaller nucleus pulposus regions and irregular endplates.^[^ ^54^ ^]^ We noticed that hChon4 also preferentially expresses *CCN2* (Table S7, Supporting Information), and neonatal *Ccn2‐GFP* mice showed that Ccn2 was expressed in the area where the EP and growth plate form later as well as in the ossification center and NP (Figure 4E and Figure S9D, Supporting Information). In addition, the loss of *Adgrg6* was reported to cause endplate‐driven herniation in mice.^[^ ^55^ ^]^ hChon4 also expresses markers of human skeletal stem cells, such as *THY1 (CD90)*, *PDPN*, and *CD164* (Figure 4F), since PDPN^+^CD146 (MCAM)*^−^* CD73^+^CD164^+^ was reported to mark human skeletal stem cells.^[^ ^56^ ^]^ Meanwhile, hChon4 exhibits strong enrichment of *EBF1*, *NFAT5*, and *SOX9* (Figure 4D). SOX9 is a pivotal transcriptional factor in chondrocytes, and NFAT5 contributes to IVD embryogenesis.^[^ ^57^ ^]^ Notably, the expression of *CTSK* was quite low in hChon4 (Figure 4F), while Ctsk^+^ lineage cells did not comprise AF and EP cells in the mouse postnatal IVD (Figure 3H). Thus, these data suggest that hChon4 is involved in EP and IAF development, and it might be a kind of chondrogenic progenitor cell.

The pseudotime analysis revealed a convergent trajectory with the developmental one (Figure 4G). hChon1 was mainly distributed at the start, which was consistent with the RNA velocity analysis (Figure S9E, Supporting Information). The trajectory appeared to develop into two branches, hChon3 (regulating endochondral ossification) and hChon4 (regulating EP/IAF development) (Figure 4G). The chondrogenic matrisome signatures, including *COL2A1*, *ACAN*, *COMP*, and *HAPLN1*, increased along both branches (Figure 4H). More specifically, the expressions of *COL1A1*, *VEGFA*, and *HHIP* increased along the ECO trajectory, while those of *MATN1*, *GDF5*, *CSGALNACT1*, and *CYTL1* increased along the EP/IAF trajectory (Figure 4H). Of note, PDPN^+^CD146(MCAM)^+^ bone/cartilage/stromal progenitors (BCSPs) were reported to differentiate into PDPN^+^CD146(MCAM)*^−^* chondroprogenitors and PDPN*^−^* CD146(MCAM)^+^ bone/stromal progenitors (BSPs).^[^ ^56^ ^]^ The expression of *MCAM* continued to increase along the ECO branch (Figure 4H), indicating that hChon3 regulates the VB ossification.

Taken together, our results temporally and functionally demonstrate the development of vertebral chondrocytes during human early IVD formation, and they further suggest that GDF5^+^CCN2^+^ hChon4 might be a human EP/IAF progenitor.

### 2.5 Temporal Characterization of the Developing Outer AF (OAF)

**Figure 5:**
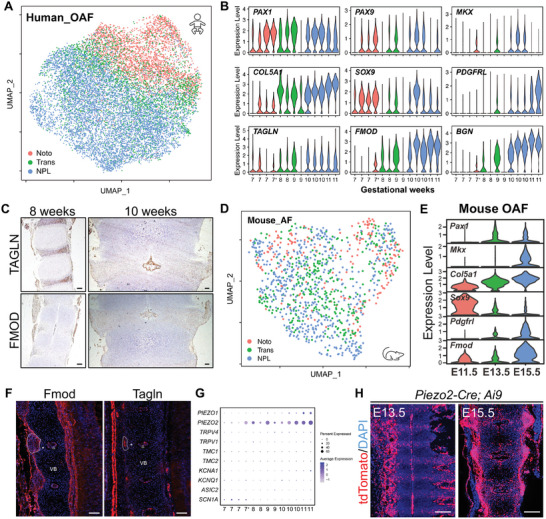
Characterization of OAF cells during early IVD formation. A) UMAP visualization of the OAF cluster during human early IVD formation at the Noto, Trans, and NPL stages. B) Representative violin plots showing the expressions of *PAX1*, *PAX9*, *MKX*, *COL5A1*, *SOX9*, *PDGFRL*, *TAGLN*, *FMOD*, *and BGN* during human early IVD formation. C) Representative IHC images of TAGLN (upper) and FMOD (lower) in human axial skeleton sections at the indicated developmental stages. Scale bar, 100 µm. D) UMAP visualization of the OAF cluster during mouse early IVD formation at the Noto, Trans, and NPL stages. E) Representative violin plots showing the expressions of *Pax1*, *Mkx*, *Col5a1*, *Sox9*, *Pdgfrl*, and *Fmod* during mouse early IVD formation. F) Representative IF images of Fmod (left) and Tagln (right) in mouse axial skeleton sections at E14.5. The dotted line indicates the presumptive OAF. The asterisks indicate the presumptive IAF. Triangles indicate NP. VB, vertebral body. Scale bar, 200 µm. G) Dot plot showing the mean expression of selected mechanosensitive ion channel genes in the human OAF along with early IVD formation. Dot size indicates the percentage of cells with detected expression. H) Representative images of lineage tracing in *Piezo2‐Cre;Ai9* mouse IVDs. Scale bar, 200 µm.

We found that cluster 6 (in hCT) highly expressed *PAX1* and *PAX9* through all three stages and increased the expressions of *MKX* and *COL5A1* (**Figure** **5**A,B and Figure S10A, Supporting Information). *PAX1/9* are known to be downregulated once prechondrogenic cells mature into chondrocytes and they persist to be expressed in OAFs to regulate their development.^[^ ^58^ ^]^ *MKX* was reported to be preferentially expressed in OAF and to promote its maintenance and regeneration.^[^ ^59^ ^]^ Thus, we defined cluster 6 as the developing OAF. This OAF also showed increased expression of other AF markers, such as *TAGLN*, *FMOD*, and *BGN* (Figure 5B,C). Compared to hChon4, which is supposed to regulate IAF development, this OAF showed reduced expression of *CCN2* (Figure S10B, Supporting Information). In mice, we found that cluster 4 preferentially expressed *Pax1*, *Mkx*, and *Pdgfrl* (Figure 5D,E), and defined it as the developing AF in mice. The expressions of Tagln and Fmod in mouse OAFs were also validated by immunostaining (Figure 5F).

As mentioned above, IVD is essential for mechanical stabilization of the axial skeleton. We next focused on the expressions of different mechanosensitive ion channels in the developing OAF at the different stages. We found that the expression of *PIEZO2* drastically increased with OAF development (Figure 5G). Then, when the *Piezo2‐EGFP‐IRES‐Cre* mouse was crossed with a Rosa‐tdTomato reporter mouse (*Ai9*) to perform lineage tracing, we found that the tdTomato^+^ Piezo2 lineage cells (Piezo2^+^ cells and their descendants) included some NC/NP cells and most AF cells (Figure 5H), since the EGFP signal was too weak to be reliably detected.^[^ ^60^ ^]^ These results indicate a potential role of PIEZO2 in AF biomechanics.

Taken together, our results characterize the temporal changes of the mesenchyme‐derived OAF, which differ from those of the chondrogenic IAF, during early IVD formation.

### 2.6 Signaling Network for the Intercellular Crosstalk between Developing NC/NP Cells and Vertebral Chondrocytes during Early Human IVD Formation

**Figure 6:**
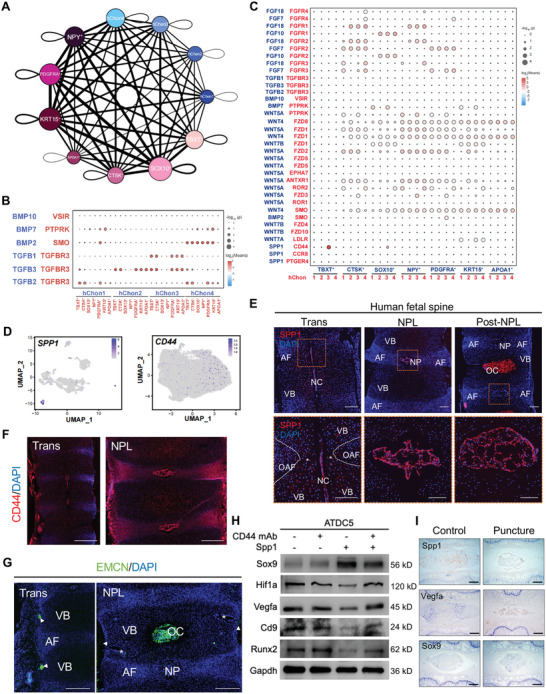
Overview of the crosstalk networks between developing NC/NP and vertebral chondrocytes during early IVD formation. A) Overview of the cellular network between developing NC/NP cells and vertebral chondrocytes during human early IVD formation. Dots indicate cell subpopulations. The dot size indicates the relative quantity of each subpopulation. The thickness of the directed line indicates the relative quantity of significant ligand–receptor pairs between any two pairs of subpopulations. B) Dot plot showing the communication probability of the indicated ligand–receptor pairs between four vertebral chondrocyte subclusters (sending signals) and seven NC/NP subclusters (accepting signals). C) Dot plot showing the communication probability of the indicated ligand–receptor pairs between seven NC/NP subclusters (sending signals) and four vertebral chondrocyte subclusters (accepting signals). D) UMAP plots showing the expression of *SPP1* and *CD44* on the UMAP. E) Representative IF images of SPP1 in human axial skeleton sections at the indicated developmental stages. Orange dotted squares are magnified under each image. The white dotted line indicates the presumptive OAF. The asterisks indicate the presumptive IAF. AF, annulus fibrosus; OAF, outer annulus fibrosus; IAF, inner annulus fibrosus; VB, vertebral body; NC, notochord; NP, nucleus pulposus; OC, ossification center. Scale bar, 100 µm. F) Representative IF images of CD44 in human axial skeleton sections at the indicated developmental stages. Scale bar, 500 µm. G) Representative IF images of EMCN in human axial skeleton sections at the indicated developmental stages. The arrows indicate the immunofluorescent signals surrounding the VB. The asterisks indicate the cartilage canals. AF, annulus fibrosus; VB, vertebral body; NP, nucleus pulposus; OC, ossification center. Scale bar, 500 µm. H) Western blot results showing the expression of Vegfa, Cd9, Hif1a and Sox9 in ATDC5 chondrogenic progenitor cells after rSpp1 treatment. I) Representative IHC images of Spp1 in control and degenerated rat IVDs. Scale bar, 200 µm.

Notochordal signaling is essential for VB segmentation and formation,^[^ ^61^ ^]^ so we next investigated the cellular interactions among NC/NP cells and vertebral chondrocytes using CellPhoneDB.^[^ ^62^ ^]^ Relative active bidirectional interactions among these 11 clusters showed highly regulated cellular communications (**Figure** **6**A) in which BMP, WNT, FGF, and TGF‐*β* pathways were vital signaling pathways regulating early IVD formation (Figure S11A, Supporting Information).

First, we determined that TGF‐*β*2 (mainly secreted by hChon1 and hChon4) and TGF‐*β*3 (mainly secreted by hChon2 and hChon3) modulated matrisome clusters (CTSK^+^, TBXT^+^, and PDGFRA^+^) via *TGFBR3* (Figure 6B). We also found that hChon3 modulated the developing NC/NP cells via *TGFB1–TGFBR3* (Figure 6B). The TGF‐*β* family is essential for the proper formation and ECM metabolism of IVD. Specifically, *Tgfbr2* conditional knockout mice displayed incomplete and even missing IVDs without affecting vertebral chondrocyte differentiation,^[^ ^63^ ^]^ and a recent study showed that TGF‐*β*3 treatment in vitro enhanced the chondrogenesis of adult NP progenitor cells.^[^ ^4b^ ^]^ Activating TGF‐*β* pathway by TGF‐*β*3 administration for 15 d promoted human pluripotent stem cells‐derived notochord‐like cells to differentiate into nucleus pulposus‐like cells, characterized by the expression of *ACAN* and *COL2A1*.^[^ ^36^, ^64^ ^]^ The main receiver cells of TGF‐*β* signaling were CTSK^+^, TBXT^+^, and PDGFRA^+^ subpopulations, which are defined in this study as matrisome clusters. Meanwhile, we found that the potential EP/IAF progenitors (hChon4) also modulated NC/NP via *BMP2–SMO* and *BMP7–PTPRK* (Figure 6B). BMP2/7 enhances matrix production of bovine NP cells,^[^ ^65^ ^]^ and recombinant human BMP2 is widely applied in spinal fusion in clinics.^[^ ^66^ ^]^ These data demonstrate that TGF‐*β* and BMP signaling from vertebral chondrocytes modulates the anabolism of developing NC/NP cells.

WNT4/5a secreted by developing NC/NP cells, except for TBXT^+^, modulates developing vertebral chondrocytes via Frizzled receptors (*FZD1/8*), *SMO*, and *ANTXR1* (Figure 6C). The developing NC/NP clusters, especially CTSK^+^, also secrete *FGF7/10/18* to modulate developing vertebral chondrocytes via *FGFR1/2/3* (Figure 6C). WNT and FGF are highly conserved signaling pathways required for normal skeletal development,^[^ ^67^ ^]^ and conditioned medium from notochordal cells was reported to enhance the chondrogenesis of human mesenchymal stem cells, without demonstration of soluble factors.^[^ ^68^ ^]^

Of note, we observed that *SPP1* secreted by developing NC/NP cells, especially TBXT^+^, modulated hChon3 via *CD44* (Figure 6C). Previous bioinformatic analysis reported that *SPP1* is involved in the interaction between NP progenitor cells and EP stromal cells at the adult stage,^[^ ^4b^ ^]^ but no direct evidence has been reported yet. We found that *SPP1* is expressed in human developing NC/NP cells as well as in the VB ossification center since *SPP1* is a known osteogenic marker (Figure 6D,E). In addition to being a well‐known marker for stem cells, CD44 plays important roles in various physiological processes, including limb development, and chondrocytes with greater CD44 expression show higher chondrogenic capacity.^[^ ^69^ ^]^ Here, we found that CD44 was ubiquitously expressed in the human fetal spine, including preferentially in the NP, AF, and VB ossification centers (Figure 6D,F). Meanwhile, we found that blood vessels, marked by the expression of EMCN, predominantly lined the VB instead of the NP or AF (Figure 6G and Figure S11B, Supporting Information). These results prompted us to investigate the role of SPP1 in regulating chondrogenesis. Recombinant mouse Spp1 (rSpp1) treatment significantly upregulated the expression of the master regulator for chondrogenesis (Sox9^[^ ^70^ ^]^) and decreased the expressions of key regulators of vascularization (Hif1a, Vegfa^[^ ^71^ ^]^) and hypertrophic markers (Runx2 and Cd9^[^ ^72^ ^]^) in non‐induced ATDC5 chondrogenic progenitor cells after three days (Figure 6H and Figure S11C, Supporting Information). However, the administration of CD44 monoclonal antibodies blocked these changes (Figure 6H and Figure S11C, Supporting Information). We further found that the expression of Spp1 was drastically downregulated, while the expressions of Vegfa and Col10a1 (hypertrophic marker^[^ ^73^ ^]^) significantly elevated in the puncture‐induced IVDD rat model (Figure 6I and Figure S11D, E, Supporting Information). These results demonstrate that notochordal SPP1 might suppress hypertrophy and vascularization while stimulating chondrogenesis by interacting with *CD44*.

Notably, SOX10^+^ also interacted with hChon3 via *SPP1–CD44* (Figure 6C). Meanwhile, we found that *SEMA3E*, which is secreted by the NC and required for the maintenance of the avascular midline,^[^ ^74^ ^]^ was preferentially expressed in SOX10^+^ cells (Figure S11F, Supporting Information). *SEMA3A*, another semaphorin that might repel neuronal and vascular ingrowth in the healthy NP,^[^ ^75^ ^]^ was preferentially expressed in SOX10^+^ and CTSK^+^ subpopulations, and the expression of *SEMA3A* in CTSK^+^ increased with IVD development (Figure S11F, Supporting Information). These results indicate the potential role of SOX10^+^ and CTSK^+^, in addition to that of TBXT^+^, which maintained the avascular microenvironment within developing NC/NP.

Taken together, our results revealed the existence of complicated cellular crosstalk with a hierarchical signaling pathway between developing NC/NP cells and vertebral chondrocytes and further demonstrated that notochordal signaling, including SPP1 and semaphorins, is vital for IVD formation and health.

### 2.7 Characterization of the Connective Tissue Subclusters in Humans and Mice

**Figure 7:**
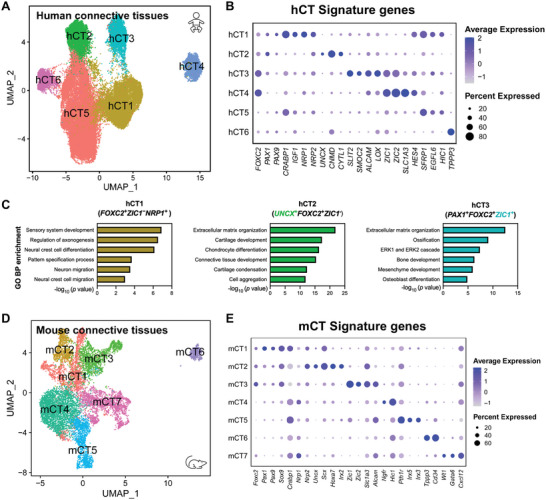
Spatial transcriptome analysis of developing IVD cells. A) UMAP visualization of the six connective tissue subclusters during human early IVD formation at the Noto, Trans, and NPL stages. B) Dot plot revealing the expressions of signature genes among the six human connective subclusters. C) Representation analysis of GO categories showing different functions for the hCT1 (left), hCT2 (middle), and hCT3 (right) clusters. D) UMAP visualization of the seven connective tissue subclusters during mouse early IVD formation at the Noto, Trans, and NPL stages. E) Dot plot revealing the expressions of signature genes among the seven mouse connective subclusters.

In addition to the hOAF (cluster 6), clusters 11 and 14 in hCT were identified as tenocytes (hTN) according to their expressions of *TNMD* and *SCX* (Table S9, Supporting Information). We further divided the remaining hCT into six subpopulations, as follows: hCT1 (*FOXC2^+^ZIC1^−^NRP1^+^*, clusters 8, 16, and 18), hCT2 (*UNCX^+^FOXC2^+^ZIC1^−^*, cluster 10), hCT3 (*PAX1^+^FOXC2^+^ZIC1^+^*, cluster 12), hCT4 (*PAX1^−^FOXC2^+^ZIC1^+^*, clusters 15 and 19), hCT5 (*FOXC2^−^ZIC1^+^*, clusters 7, 9, and 13), and hCT6 (*TPPP3^+^PDGFRA^+^*, cluster 17) (**Figure** **7**A,B and Figure S12A, Supporting Information). *Tppp3^+^Pdgfra^+^* cells were recently demonstrated to be tendon stem cells contributing to tendon regeneration.^[^ ^76^ ^]^ This indicated that hCT6 might be a population of tendon stem cells. hCT1‐3 were identified as mesenchymal sclerotomes according to the expressions of *FOXC2* and *PAX1* (Figure 7B), which are known to develop into VBs and AF tissue.^[^ ^77^ ^]^ Sclerotomal hCT1 preferentially expressed *CRABP1, NGFR*, and *NRP1* (Table S10, Supporting Information), and GO analysis revealed that hCT1 is enriched in “neuron migration” and “neural crest cell migration” (Figure 7C). The anterior half‐sclerotome was reported to express *CRABP1*, *NRP1*, and NRP2, and it regulates neural crest cell migration and neurite outgrowth.^[^ ^78^ ^]^ This suggested the sclerotomal hCT1 to be the anterior half‐sclerotome. hCT2 preferentially expresses *UNCX* (Figure 7B and Table S10, Supporting Information), a marker of the posterior half‐sclerotome, which develops into the rostral VB and whole AF.^[^ ^79^ ^]^ The characteristic genes of hCT2 were enriched in “Cartilage condensation” and “Chondrocyte differentiation” (Figure 7C). The sclerotomal hCT3 preferentially expressed *ZIC1* and *ZIC2* (Figure 7B and Table S10, Supporting Information). *ZIC1* is expressed in the dorsal sclerotome, while *PAX1* is mainly expressed in the ventral sclerotome.^[^ ^80^ ^]^ It was reported that *Zic1^−/−^* mice showed developmental defects in vertebral arches and ribs rather than VBs and transverse processes.^[^ ^80^ ^]^ This indicates that hCT3 might be the dorsal mesenchymal sclerotome. Furthermore, the pseudotime analysis revealed that hCT2 subpopulation developed into hOAF and hChon4 subpopulations (Figure S12B, Supporting Information).

We next divided the mCT into seven subpopulations (Figure 7D,E; Figure S12C and Table S11, Supporting Information): mCT1, marked by the expressions of *Pax1* and *Pax9*; mCT2, marked by the expressions of *Uncx*, *Nrp1*, *Nrp2*, and *Scx*; mCT3, marked by the expressions of *Zic1* and *Zic2*; mCT4, marked by the expressions of *Ngfr* and *Hic1*; mCT5, marked by the expressions of *Pth1r*, *Irx5*, and *Irx3*; mCT6, marked by the expressions of *Tppp3* and *Cd34*; mCT7, marked by the expressions of *Wt1*, *Gata6*, and *Cxcl12*. Similarly, mCT1‐3 subpopulations were identified as mesenchymal sclerotome according to the expressions of *Foxc2*, *Pax1*, and *Pax9*, and mCT6 subpopulation was identified as tendon stem cells according to the expressions of *Tppp3* and *Cd34*.

### 2.8 Spatiotemporal Characterization of Developing IVD Cells by Spatial Transcriptome Analysis

To better characterize the spatial distribution of developing IVD cells, we performed spatial transcriptome analysis using 10x Genomics visium. The clusters were defined morphologically. At E11.5 and E12.5, the developing axial skeleton included two main subclusters: dense Chon1 and loose Chon2 (Figure S13A, Supporting Information). Chon1 was marked by the expressions of *Pax1*, *Uncx*, *and Pax9* (Tables S12–S, 14, Supporting Information) and was the posterior half‐sclerotome derivate. Conversely, loose Chon2 was marked by the expressions of *Dcn* and *Igfbp2* (Tables S12–S, 14, Supporting Information). At E13.5, the defined AF highly expressed *Pax1, Bgn*, and *Tgfbi*, while Chon preferentially expressed *Matn1*, *Comp*, and *Mia* (Tables S12–S, 14, Supporting Information). We found that *Pax1* was mainly expressed in the ventral axial skeleton, while *Zic1* was preferentially expressed in the dorsal region as well as in the spinal cord (Figure S13B, Supporting Information), which was consistent with our abovementioned results. Meanwhile, *HOX* genes are known to specify the morphological identity of the vertebrae and spinal cord,^[^ ^81^ ^]^ and we determined that *Hox10/11* genes were predominantly expressed in the caudal region, while *Hox4/5* were preferentially expressed in the rostral region (Figure S14, Supporting Information).

Although the resolution of the 10x Genomics visium was not ideal for capturing developing NC/NP cells (E11.5, 4 spots; E12.5, 8 spots; and E13.5, 14 spots), we succeeded in determining their signature genes (Tables S12–S, 14, Supporting Information), which was in line with our abovementioned data. Specifically, these NC clusters preferentially expressed canonical notochord markers (*Car3*, *T*, *Shh*, *Krt19*, and *Krt18*) as well as novel markers, including *Spp1* and *Sox10* (Figure S13C,D, Supporting Information). Low expression of *Ctsk* was detected in developing NC/NP cells from E11.5 to E13.5 (Figure S13E, Supporting Information). Interestingly, we found that a previously reported NC/NP marker, *Lepr*, was preferentially expressed at E11.5 and E12.5 and decreased with IVD development in mice (Figure S13F,G, Supporting Information), but substantially low expression of *LEPR* was observed in developing human NC/NP cells (Figure S13G, Supporting Information), thus indicating the existence of cellular divergence between humans and mice during early IVD formation.

Taken together, our data describe the spatiotemporal distribution of early IVD cells and sclerotomal mesenchymal cells, and they were partially validated by spatial transcriptome analysis during early IVD formation.

## 3 Discussion

In this study, we generated a large scRNA‐seq dataset, which included a total of 177 725 cells from 13 human fetuses at different gestational weeks to investigate key signaling events underlying human early IVD formation. Samples of the human embryonic axial skeleton ranging from seven to 11 gestational weeks of age were included in this scRNA‐seq dataset and further grouped into three developmental stages morphologically. NC cells are thought to disappear starting in human adolescent NPs,^[^ ^3^ ^]^ although this has been questioned by recent studies.^[^ ^4^, ^82^ ^]^ Furthermore, the loss of NC cells from the NP was reported to be closely associated with IVDD onset,^[^ ^68^ ^]^ and NC cells are known to regulate IVD homeostasis.^[^ ^83^ ^]^ The majority of recent investigations have focused on identifying new NP stem/progenitor cells in the adult mature IVD,^[^ ^4^, ^8^ ^]^ and some scholars have described the cellular heterogeneity inside the human adult IVD.^[^ ^8b^ ^]^ Here, we focused on the NC‐to‐NP transition instead of mature adult IVDs and resolved the spatiotemporal cellular diversity at the single‐cell level using transcriptomic profiling in the human embryonic axial skeleton.

We identified two novel markers (*SOX10* and *CTSK*) of developing NC/NP cells in humans and mice, which were then validated via lineage tracing. In addition, *SPP1* and *KRT15* are novel NC/NP markers in both humans and mice but require further genetic validation. Furthermore, we analyzed two published scRNA‐seq datasets of human adult IVDs (GSE165722 and GSE160756; Figure S15A,B, Supporting Information), and checked the expression of hNC signature genes (*CTSK, TBXT, SOX10, APOA1, KRT15, PDGFRA*, and *NPY*) (Figure S15A–D, Supporting Information). The early hNC markers, such as *SOX10*, *NPY*, and *APOA1*, were hardly expressed in both nondegenerative (GSE160756) and degenerative (GSE165722) NP tissues. *TBXT* was expressed in the Notochord cluster within nondegenerative NP tissues, while *CTSK* and *PDGFRA* were expressed in both nondegenerative and degenerative NP tissues. We then integrated our hNC data with these two datasets (Figure S15E, Supporting Information). The TBXT^+^, CTSK^+^, and PDGFRA^+^ subpopulations were distributed closely alongside adult IVD clusters in the integrated UMAP, while the KRT15^+^ and SOX10^+^ subpopulations were not. This trend suggests that the TBXT^+^, CTSK^+^, and PDGFRA^+^ subpopulations might be still retained in adult stage of IVD development.

To achieve NP‐specific genetic experiments, several *Cre‐*expressing mouse strains have been generated thus far.^[^ ^8^, ^68^, ^84^ ^]^ The *Shh*‐*Cre* strain was the first reported and most used NC‐*Cre* mouse,^[^ ^85^ ^]^ but the tamoxifen inducible *Shh*‐*CreER^T2^* strain failed to target the postnatal NP,^[^ ^31^ ^]^ suggesting that *SHH* is an early NC marker. In our study, we found that the *Sox10‐Cre* and *Sox10‐CreER^T2^* strains showed similar cellular and temporal spectra to those of the *Shh‐Cre* and *Shh‐CreER* ^T2^ strains. In addition, our lineage tracing results confirmed that *Ctsk‐Cre* is suitable for NP‐specific genetic experiments, since Ctsk^+^ lineage cells were located in the NP region instead of the AF, EP, or growth plate within the postnatal IVD (Figure 3H). *Lepr‐Cre* and *Uts2r‐CreER^T2^* strains were recently reported to target NP cells in mice,^[^ ^8^, ^84^ ^]^ thus providing more NC‐Cre strain choices. The Lepr^+^ lineage cells included most NC cells, as shown by *Lepr‐Cre;Ai9* mice at E12.5,^[^ ^84^ ^]^ and we also found that *Lepr* is highly expressed in the mouse T^+^. However, the expression of *LEPR* was quite low in human embryonic NC/NP cells. The expression of *UTS2R/Uts2r* was hardly detected in either our human or mouse embryonic datasets, which might be because *Uts2r* is a postnatal NP stem/progenitor marker.^[^ ^8a^ ^]^ In addition, NPY^+^, PDGFRA^+^, and APOA1^+^ clusters were uniquely defined in humans, while Rprm^+^, Tcf21^+^, and Adipoq^+^ clusters were unique in mice. However, canonical NC/NP markers, including *TBXT*, *CA3*, *SHH*, *KRT19*, *KRT18*, and *KRT8*, were detected in both humans and mice. Our results suggest the existence of cellular and molecular diversity in developing NC/NP cells between humans and mice, which provided comparable human and mouse datasets to help with NC/NP‐specific genetic experiments.

In addition to being a classical marker of osteoclasts, CTSK was recently proven to be a marker of periosteal stem cells, perichondrial progenitors and tendon progenitors.^[^ ^86^ ^]^ We defined CTSK^+^ as a matrisome cluster present in both human and mouse developing NC/NP cells and validated its spatiotemporal dynamics by lineage tracing. There has historically been a hypothesis that cartilage‐like cells within the NP originate from the surrounding EP or the perichondrium at the periphery of the IVD.^[^ ^87^ ^]^ Although CTSK was reported to be a marker of periosteal stem cells and perichondrial progenitors in long bones,^[^ ^86a^ ^]^ our results showed that Ctsk^+^ lineage cells are distributed within the NP instead of the AF, EP, or growth plate, suggesting that these Ctsk^+^ cells to be of notochordal origin. These findings coincide with another theory that, during normal development and aging, all cells within the NP are NC‐derived.^[^ ^88^ ^]^ Tu et al. suggested that *THY1^+^* (*CD90^+^*) fibroNPCs are the progenitor cells, which could be considered as a potential therapeutic target of degenerative spinal diseases.^[^ ^8b^ ^]^ They supposed that fibroNPCs may differentiate into adhesion NPCs and other NPCs subsets, and homeostatic NPCs were mainly distributed at the end. We further divided the fibroNPCs into two subsets, the CTSK^High^THY1^High^ and the CTSK^Low^THY1^Low^ clusters (Figure S15F–H, Supporting Information). Monocle analysis revealed that the CTSK^High^THY1^High^ cluster mainly existed at the start of the fibroNPCs trajectory (Figure S15I, Supporting Information). Furthermore, the CTSK^+^ subpopulation also highly expressed *THY1* (*CD90*) (Figure S15J, Supporting Information). Taken together, the late embryonic CTSK^+^ subpopulation might be retained at adult stage and give rise to adult fibroNPCs.

The IVD is constantly subjected to various mechanical forces, and its exposure to excessive mechanical stress is known to cause IVDD.^[^ ^89^ ^]^ At an early stage, the rising osmotic pressure permits the NC to elongate,^[^ ^90^ ^]^ while, in mature healthy NPs, hydrostatic and osmotic pressures are altered in response to external loading conditions.^[^ ^91^ ^]^ Negatively charged proteoglycans and other ECM components provide high osmotic pressure to maintain a hydrated structure within the mature IVD. In this study, we found that mechanosensitive channels, including *PIEZO1*, *PIEZO2*, and *TRPV4*, were preferentially expressed in matrisome NC/NP clusters, such as TBXT^+^ and CTSK^+^ clusters. TRPV4 was the first osmotically sensitive vertebrate ion channel to be discovered,^[^ ^92^ ^]^ and it has been found to be expressed in a variety of tissues.^[^ ^93^ ^]^ TRPV4 was activated by hypo‐osmotic cell swelling, and its activation promotes chondrogenesis by inducing *SOX9* transcription.^[^ ^94^ ^]^ Recently, the expression of *Trpv4* in the NP and IAF at the embryonic stage was validated by the *Trpv4^Lacz/WT^* strain, and Trpv4‐dependent Ca^2+^ responses regulate the expression of *Acan* and *Prg4* in AF cells.^[^ ^95^ ^]^ It was also reported that a low magnitude of compression promoted the biosynthesis of mesenchymal stem cells toward NP cells via TRPV4.^[^ ^96^ ^]^ However, the function of TRPV4 in the NC‐to‐NP transition has not yet been illustrated. Because TBXT^+^ is enriched in the expression of proteoglycans and preferentially expresses *TRPV4*, the potential role of TRPV4 in maintaining osmotic pressure in the NC/NP deserves more investigation. PIEZO1 could also be gated by osmotic pressure,^[^ ^97^ ^]^ and conditional knockout of Piezo1 leads to overhydrated red blood cells with increased osmotic fragility.^[^ ^98^ ^]^ However, gain‐of‐function mutations in *PIEZO1* are associated with dehydrating red blood cell disease xerocytosis.^[^ ^99^ ^]^ We previously reported that ECM stiffness accelerated the degeneration of adult NP cells by activating PIEZO1,^[^ ^89^ ^]^ but understanding the role of PIEZO1, which regulates osmotic pressure in embryonic NC/NP cells, necessitates further experimental evidence. CTSK^+^ is associated with the “response to mechanical stimulus” and preferentially expresses *PIEZO2*. However, little is known about the function of PIEZO2 in IVD development and degeneration, despite its expression in both NP and AF.

A healthy adult IVD is largely avascular and aneural with sparse innervation and vascularization to the peripheral AF and EP.^[^ ^100^ ^]^ Neoinnervation and neovascularization are integral steps in the pathogenesis of IVDD, especially the ingrowth of nociceptive nerve fibers, which might be associated with chronic low back pain.^[^ ^101^ ^]^ Evidence suggests that proteoglycans, such as ACAN, inhibit neural and vascular ingrowth.^[^ ^102^ ^]^ In addition, SEMA3A may also act as a barrier to neuronal ingrowth within healthy IVDs.^[^ ^75^ ^]^ However, few additional studies to date have investigated other inhibitory factors on blood vessels in healthy IVDs. Our study revealed that the SOX10^+^, TBXT^+^, and CTSK^+^ clusters may be vital for the maintenance of the avascular midline during IVD formation. Furthermore, we found that rSpp1 enhanced chondrogenesis and inhibited the expression of Vegfa in chondrogenic progenitor ATDC5 cells. These results indicate that SPP1 is an inhibitory factor influencing vascular ingrowth during IVD development. We further found that the expression of Spp1 was drastically decreased in degenerated NPs (Figure 6I), thus suggesting a potential therapeutic role of SPP1 addition in IVDD.

We found that CD44 is ubiquitously expressed in the human fetal spine, which inspired us to explore the potential roles of *SPP1‐CD44* in different IVD cells. Human primary nucleus pulposus cells (hNPs), rat annulus fibrosus cells (rAFs), and rat endplate chondrocytes (rEPs) were isolated and cultured. First, the administration of rSpp1 in hNP cells decreased the expressions of hypertrophic markers (CD9 and RUNX2) and vascularization markers (HIF1A and VEGFA) (Figure S16A, Supporting Information). However, rSpp1 treatment seemed to have no effect on chondrogenesis in hNP cells. CD44 mAb treatment could reverse the reduced expression of CD9 and VEGFA induced by rSpp1. Second, neither rSpp1 nor CD44 mAb treatment had effects on rAF cells (Figure S16B, Supporting Information). At last, rSpp1 treatment in rEP cells stimulated chondrogenesis, which was reversed by CD44 mAb treatment (Figure S16C, Supporting Information). rSpp1 treatment also downregulated the expression of vascularization markers (Hif1A and Vegfa), but CD44 mAb did not reverse the tendencies. Taken together, SPP1 may play different roles in different IVD cells not only through interacting with CD44, although CD44 is ubiquitously expressed in the human fetal spine.

The hChon4 (expressing *CCN2*, *GDF5*, and *PIEZO2*) is supposed to give rise to the chondrogenic EP/IAF. The *Ccn2‐GFP* mice showed that *Ccn2* was expressed in the NP, the outer layer of IAF adjacent to the OAF, and VB ossification centers at postnatal IVDs (Figure 4E and Figure S9D, Supporting Information), consistent with another paper reporting that *Ccn2‐Lacz* was expressed in the IAF and NP at E16.5.^[^ ^103^ ^]^ Thus, *Ccn2* could label both EP/IAF and NP in embryonic IVDs. Meanwhile, Christina Munndy and colleagues previously showed that Gdf5^+^ lineage cells were restricted to the AF region instead of the NP region in the intervertebral joints at P0.^[^ ^104^ ^]^ Taken together, the hChon4, marked by the expression of both *CCN2* and *GDF5*, is a chondrogenic cluster that might give rise to EP/IAF. Meanwhile, *Ctsk* was hardly expressed in the hChon4, and we also found that *Ctsk‐Cre;mT/mG* mice labeled only some NP and VB ossification center cells, rather than AF or EP cells (Figure 3H). At last, the hChon4 expressed markers of human skeletal stem cells, such as *THY1 (CD90)*, *PDPN*, and *CD164* (Figure 4F), suggesting that it might be a kind of chondrogenic progenitor cell, and more experimental evidence is still needed to prove their “stemness”.

In conclusion, we reported scRNA‐seq analysis findings of human and murine fetal axial skeletons during early IVD formation combined with NC/NP subcluster lineage tracing. We revealed the heterogeneity and spatiotemporal dynamics of axial skeleton cells and further compared the differences between humans and mice. We reported three novel NC/NP markers (*SOX10*, *CTSK* and *SPP1*) and demonstrated the diverse roles of NC/NP subpopulations during IVD formation, which may be leveraged to develop preventative and regenerative strategies for degenerative spinal diseases.

## 4 Experimental Section

### Clinical Specimen

This study protocol was approved by the ethics committee of the First Affiliated Hospital of Sun Yat‐sen University (Nos. [2015]76 and [2020]320). All of the donors signed written informed consent for the use in research of fetal material arising from the termination of their pregnancy. All the donors were informed about the purpose of the research and there was no compensation offered for donation. All of the embryos (*n* = 13 for scRNA‐seq, *n* = 20 for in‐house bulk RNA‐seq and histological verification) were considered structurally normal on ultrasound examination before termination.

Each sample was proceeded and microdissected by a spine surgeon with the assistance of a senior obstetrician under a stereo microscope, following the standard operating procedure. Briefly, the whole axial skeleton and their adjacent tissues (ligaments, ribs and spinal cord) were carefully dissected from the embryo, followed by removal of the ribs (at their costovertebral joints), the spinal cord and the vertebral arches by using microsurgical forceps and scissors. The vertebral column order was recognized by the ribs. At last, the anterior and posterior longitudinal ligaments were gently separated from the spine. The resulting whole fetal axial skeleton was then cut into pieces, followed by trypsin (3 mg mL^−1^, 37 °C, 10 min) and type I collagenase (5 mg mL^−1^ in RPMI, 37 °C, 60 min) digestion. The filtered (70 µm) cell suspension was then treated with red blood cell lysis buffer to remove red blood cells. The cells were washed twice with wash solution (0.01 × 10^−3^ m EDTA and 0.04% BSA in PBS) and resuspended in PBS followed by filtration (40 µm).

### scRNA‐seq

scRNA‐seq was performed by Guangzhou Gene Denovo Honour Biotechnology Co., Ltd., and a 10x Genomics GemCode single‐cell instrument was used to generate single‐cell Gel Bead‐In‐EMlusion (GEMs). Single‐cell capture libraries were constructed with Chromium Next GEM Single Cell 3’ Reagent Kits v3.1 according to the manufacturer's instructions. The remaining biochemical reagents and primers in the post‐GEM reaction mixture were removed with silane magnetic beads. R1 (the read 1 primer sequence) was added during GEM incubation, while P5, P7, a sample index, and R2 (the read 2 primer sequence) were added during library construction through end repair, A‐tailing, adaptor ligation, and PCR. The final libraries contained the P5 and P7 primers used in Illumina bridge amplification. The Single Cell 3’ Protocol produced Illumina‐ready sequencing libraries.

### Single‐Cell RNA Statistical Analysis

Conversion, alignment, and count quantification were performed with the 10x Genomics Cell Ranger software (version 3.1.0). After filtering out reads with low‐quality barcodes and unique molecular identifiers (UMIs), reads uniquely mapped to the transcriptome and intersecting an exon at least 50% were considered for UMI counting. Before quantification, the UMI sequences were corrected for sequencing errors, and valid barcodes were identified based on the EmptyDrops method.^[^ ^105^ ^]^ The cell‐by‐gene matrices were produced via UMI counting and cell barcode calling. The Seurat package (version 4.1.0) was used for cell normalization and regression based on the expression table according to the UMI counts of each sample and the percentage of mitochondria rate to obtain the scaled data. The Harmony package (https://github.com/immunogenomics/harmony)^[^ ^106^ ^]^ was applied to correct the batch effect followed by UMAP reduction construction. PCA was constructed based on the scaled data, and the top 2000 highly variable genes were used for UMAP construction. Using the graph‐based cluster method (resolution, 0.9), the unsupervised cell cluster result was acquired, and the marker genes were calculated by the FindAllMarkers function under the following criteria: log_2_FC > 0.75, adjusted *P*‐value < 0.01, and pct.1 > 0.25. To identify the cell type details, clusters of the same cell type were selected for re‐UMAP analysis, graph‐based clustering, and marker gene analysis.

### GSVA

GSVA was performed by using a collection of gene sets from MSigDB (https://www.gsea‐msigdb.org/gsea/msigdb) to identify pathways and cellular processes enriched in different clusters based on the cluster‐averaged log‐transformed expression matrix.

### SCENIC Analysis

SCENIC was used to identify vertebral chondrocyte‐specific gene regulatory networks. A log‐normalized expression matrix generated using Seurat was used as input. The gene co‐expression network was identified using GENIE3. Regulons were identified via RcisTarget. The activity of each regulon for each single cell was determined via the AUC scores using the AUCell R package.

### Pseudotime Analysis

Single‐cell trajectory analysis was applied using Monocle (http://cole‐trapnell‐lab.github.io/monocle‐release/) with DDR‐Tree and default parameters. Prior to Monocle analysis, marker genes of the Seurat clustering result and raw expression counts of the cells that passed filtering were selected. Based on pseudotime analysis, branch expression analysis modeling was applied for branch fate‐determined gene analysis.

### Cell Communication Analysis

CellPhoneDB was used to analyze the expression abundance of ligand–receptor interactions between two cell types on the basis of the expression of a receptor by one cell type and a ligand by another cell type. Only receptors and ligands with expression of >10% in the specific cluster were considered for the analysis. Ligand–receptor pairs with *P*‐values > 0.05 were filtered, while the others were retained for evaluating the relationships among the different cell types.

### Differential Gene‐Expression Analysis

The function FindMarkers with the Wilcoxon rank‐sum test algorithm was used under the following criteria (unless otherwise specified in the table titles) to identify differentially expressed genes among samples: log_2_FC > 0.75, adjusted *P*‐value < 0.01, and pct.1 > 0.25.

### Mice and Treatment

All of the mice used in this study were housed in a strict pathogen‐free environment. *Sox10‐Cre* (#025807), *Sox‐CreER^T2^* (#027651), *Piezo2‐EGFP‐IRES‐Cre* (#027719), and *Ai9* (#007909) mice were originally purchased from the Jackson Laboratory (Bar Harbor, ME, USA). *Rosa26‐mTmG (mT/mG)* and wild‐type C57BL/6J mice were acquired from Prof. Ren Xu (Xiamen University). *CTSK‐Cre* mice were obtained as described in previous research.^[^ ^107^ ^]^ All of the mice analyzed were maintained on the C57BL/6 background. For lineage tracing, *Sox‐CreER^T2^*;*mT/mG* mice were generated and intraperitoneally injected with tamoxifen (20 mg mL^−1^, T5648; Sigma‐Aldrich, St. Louis, MO) in corn oil (#C8267, Sigma‐Aldrich) daily for 1 and 3 d at the indicated time points. All of the animal experiments were conducted according to guidelines approved by the institutional animal care and use committee at Xiamen University (XMULAC20190084).

### Immunohistochemical (IHC) and Immunofluorescent Assays

Tissue sections were dewaxed and rehydrated, and heat‐mediated antigen retrieval was performed. The *CCN2‐GFP* mouse sections were a kind gift from Prof. Yingzi Yang (Harvard School of Dental Medicine). The sections were incubated with primary antibodies against KRT8 (1:500, #ab53280; Abcam, Cambridge, UK), SOX10 (1:200, #ab155279; Abcam), ACAN (1:500, #ab36861; Abcam), COL1A1 (1:250, #AF7001; Affinity Biosciences, Cincinnati, OH, USA), CTSK (1:200, #ab19027; Abcam), MATN1 (1:200, #bs‐1976R; Bioss Antibodies, Woburn, MA), POSTN (1:500, #66491‐1‐Ig; Proteintech Corp., Rosemont, IL), TAGLN (1:200, #AF9266; Affinity Biosciences), FMOD (1:200, #60108‐1‐Ig; Proteintech Corp.), SPP1 (1:500, #b69498; Abcam), VEGFA (1:200, #66828‐1‐Ig; Proteintech Corp.), SOX9 (1:500, #ab185966; Abcam), and COL10A1 (1:400, #ab49945; Abcam) at 4 °C overnight. After that, the sections were incubated with HRP‐conjugated secondary antibodies, and the signals were developed with 3,3‐diaminobenzidine. The images were photographed with a microscope (BX63; Olympus Corp., Tokyo, Japan).

The frozen sections were washed with PBS followed by incubation in 0.1% Triton X‐100 for 5 min at room temperature. Then the sections were blocked with 10% goat serum for 30 min and incubated with primary antibodies against SOX10 (1:200, #ab155279; Abcam), FMOD (1:200, #60108‐1‐Ig; Proteintech Corp.), TAGLN (1:200, #AF9266; Affinity Biosciences), CD44 (1:500, #15675‐1‐AP; Abcam), SPP1 (1:500, #b69498; Abcam), and EMCN (1:500, #ab106100; Abcam) at 4 °C overnight. The next day, the sections were incubated with Alexa Fluor‐conjugated secondary antibodies for 1 h, and the cell nuclei were counterstained with DAPI. The images were photographed with a fluorescence microscope (BX63; Olympus Corp.).

### Cell Culture

The chondrogenic progenitor ATDC5 cells were cultured in DMEM:Hams F‐12 (1:1; Sigma‐Aldrich) supplemented with 1% penicillin/streptomycin (Gibco Laboratories, Gaithersburg, MD) and 10% FBS (Invitrogen, Carlsbad, CA) and cultured at 37 °C in a humidified incubator containing 5% CO_2_. ATDC5 cells were then treated with rSPP1 (200 ng mL^−1^, #763602; BioLegend, San Diego, CA) or anti‐CD44 antibodies (2.15 µg mL^−1^, #C2368; Leinco Technologies, Fenton, MO) at 37 °C for 72 h.

### Immunoblotting Analysis

Immunoblotting was performed according to standard procedures. The following primary antibodies against VEGFA (1:1000, #66828‐1‐Ig; Proteintech Corp.), CD9 (1:1000, #13403S; Cell Signaling Technology, Danvers, MA), HIF1*α* (1:250, #36169; Cell Signaling Technology), SOX9 (1:1000, #ab185966; Abcam), RUNX2 (1:1000, #12556s; Cell Signaling Technology), and GAPDH (1:5000, #10494‐1‐AP; Proteintech Corp.) were used. After incubation with HRP‐conjugated secondary antibodies for 1 h at room temperature, the signals were developed using an ECL chemiluminescence detection kit (Beyotime Biotechnology, Beijing, China) and images were captured on an ImageQuant Las4000 mini system (GE Healthcare, Chicago, IL).

### Rat Model of IVD Degeneration

The experimental procedures were approved by the institutional animal care and use committee of Sun Yat‐sen University (SYSU‐IACUC‐2021‐000973). Sprague Dawley rats were purchased from Charles River Laboratories (Beijing, China). The rat IDD model was established as described previously.^[^ ^108^ ^]^ Briefly, the rats were anesthetized, and tail skin was sterilized. In addition, needles (21‐gauge) were adopted to puncture the IVDs to a depth of ≈5 mm, rotated 360°, and kept in the IVD for 1 min. Eight weeks after the surgery, the freshly dissected rat tails were collected and fixed in 4% paraformaldehyde, decalcified in 15% EDTA, and embedded in paraffin. The sections were prepared at a thickness of 5 µm.

### Spatial Transcriptome Analysis

Spatial transcriptome analysis was performed by Shanghai OE Biotech Co. (Shanghai, China). OCT‐embedded mouse axial skeletons were cryosectioned to a thickness of 10 µm. Tissue sections were layered onto a visium spatial tissue optimization slide containing oligonucleotides for mRNA capture (10x Genomics). To synthesize fluorescently labeled cDNA, Master Mix containing reverse transcription reagents and fluorescently labeled nucleotides was added on top of the tissue sections. Fluorescent cDNA covalently linked to oligonucleotides was left on the slide after enzymatic removal of tissues. Fluorescent cDNA was visualized under fluorescence imaging conditions and verified using the visium imaging test slide. H&E and fluorescence images were compared. The permeabilization time that resulted in maximum fluorescent signals with the lowest signal diffusion was optimal. The libraries were sequenced with paired‐end 150‐bp sequencing (PE150) using the NovaSeq 6000 platform (Illumina, Carlsbad, CA). The space ranger showed the capture area of the tissue in the slide and differentiated reads for each spot based on spatial barcode information. STAR was used to assess the sample quality by the total number of spots, the number of pairs of reads in each spot, the number of detected genes, and the number of UMIs. Loupe Browser (10x Genomics) was used to visually analyze the sequencing results.

### Statistical Analysis

The results were presented as mean ± SD. Statistical significance was determined by the two‐tailed independent Student's *t*‐test for comparisons of two independent groups and by one‐way ANOVA followed by Holm–Sidak test for multiple comparisons. In all cases, a *p*‐value of less than 0.05 was considered statistically significant. All statistical analyses were conducted with the SPSS 13.0 statistical software package.

## Conflict of Interest

The authors declare no conflict of interest.

## Supporting information

Supporting Information

Supporting Information

Supporting Information

Supporting Information

Supporting Information

Supporting Information

Supporting Information

Supporting Information

Supporting Information

Supporting Information

Supporting Information

Supporting Information

Supporting Information

Supporting Information

Supporting Information

## Data Availability

The data that support the findings of this study are available from the corresponding author upon reasonable request.
