# Supplementary material for: Spatiotemporal Characterization of Human Early Intervertebral Disc Formation at Single‐Cell Resolution
Source: Adv Sci (Weinh). 2023 Mar 25;10(14):2206296. doi: 10.1002/advs.202206296 (PMC10190614; doi:10.1002/advs.202206296)
Supplement: Supplementary file 1 — Supporting Information [file ADVS-10-2206296-s010.pdf]

ADVANCED SCIENCE
Open Access

Supporting Information

for *Adv. Sci.*, DOI 10.1002/advs.202206296

Spatiotemporal Characterization of Human Early Intervertebral Disc Formation at Single-Cell Resolution

Taifeng Zhou, Yu Chen, Zhiheng Liao, Long Zhang, Deying Su, Zhuling Li, Xiaoming Yang, Xiaona Ke, Hengyu Liu, Yuyu Chen, Ricong Weng, Huimin Shen, Caixia Xu, Yong Wan, Ren Xu and Peiqiang Su**

Supporting Information

Title: Spatiotemporal characterization of human early intervertebral disc formation at single-cell resolution

Running title: scRNA-seq of human early IVD formation

Authors: Taifeng Zhou[1,#], Yu Chen[2,#], Zhiheng Liao[1,#], Long Zhang[2], Deying Su[3], Zhuling Li[1], Xiaoming Yang[1,6], Xiaona Ke[1], Hengyu Liu[1], Yuyu Chen[1], Ricong Weng[1], Huimin Shen[4], Caixia Xu[5], Yong Wan[1], Ren Xu[2,*], Peiqiang Su[1,*].

Contact information:

1. Department of Spine Surgery, Guangdong Provincial Key Laboratory of Orthopedics and Traumatology, The First Affiliated Hospital of Sun Yat-sen University, Guangzhou 510080, China.

2. State Key Laboratory of Cellular Stress Biology, Fujian Provincial Key Laboratory of Organ and Tissue Regeneration, School of Medicine, Faculty of Medicine and Life Sciences, Xiamen University, Xiamen 361102, China.

3. Guangdong Provincial Key Laboratory of Proteomics and State Key Laboratory of Organ Failure Research, School of Basic Medical Sciences, Southern Medical University, Guangzhou 510515, China.

4. Department of Gynecology and Obstetrics, The First Affiliated Hospital of Sun Yat-sen University, Guangzhou 510080, China.

5. Research Center for Translational Medicine, The First Affiliated Hospital of Sun Yat-sen University, Guangzhou 510080, China.

6. Current address: Department of Orthopedics, Renmin Hospital of Wuhan University, Wuhan 430060, China.

[#]These authors contribute equally.

***Correspondence to:**

Peiqiang Su, Department of Spine Surgery, Guangdong Provincial Key Laboratory of Orthopedics and Traumatology, The First Affiliated Hospital of Sun Yat-sen University, No.58 Zhongshan 2nd Road, Yuexiu District, Guangzhou 510080, China. Email: supq@mail.sysu.edu.cn.

Ren Xu, State Key Laboratory of Cellular Stress Biology, School of Medicine, Faculty of Medicine and Life Sciences, Xiamen University, Xiang'an South Road, Xiang'an District, Xiamen 361102, China. Email: xuren526@xmu.edu.cn.

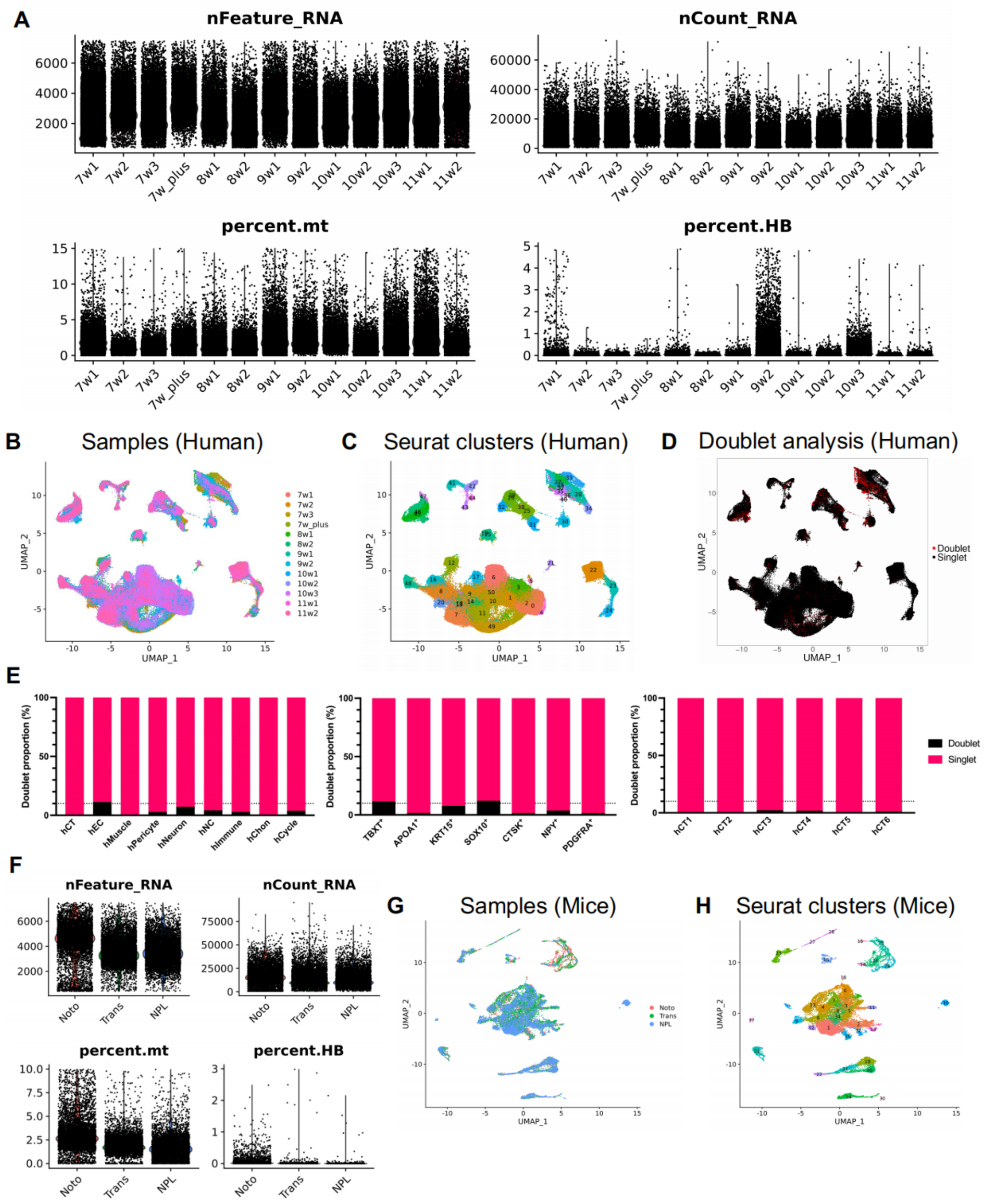

Figure S1. Single cell RNA sequencing data quality control, doublet exclusion, and batch correction.

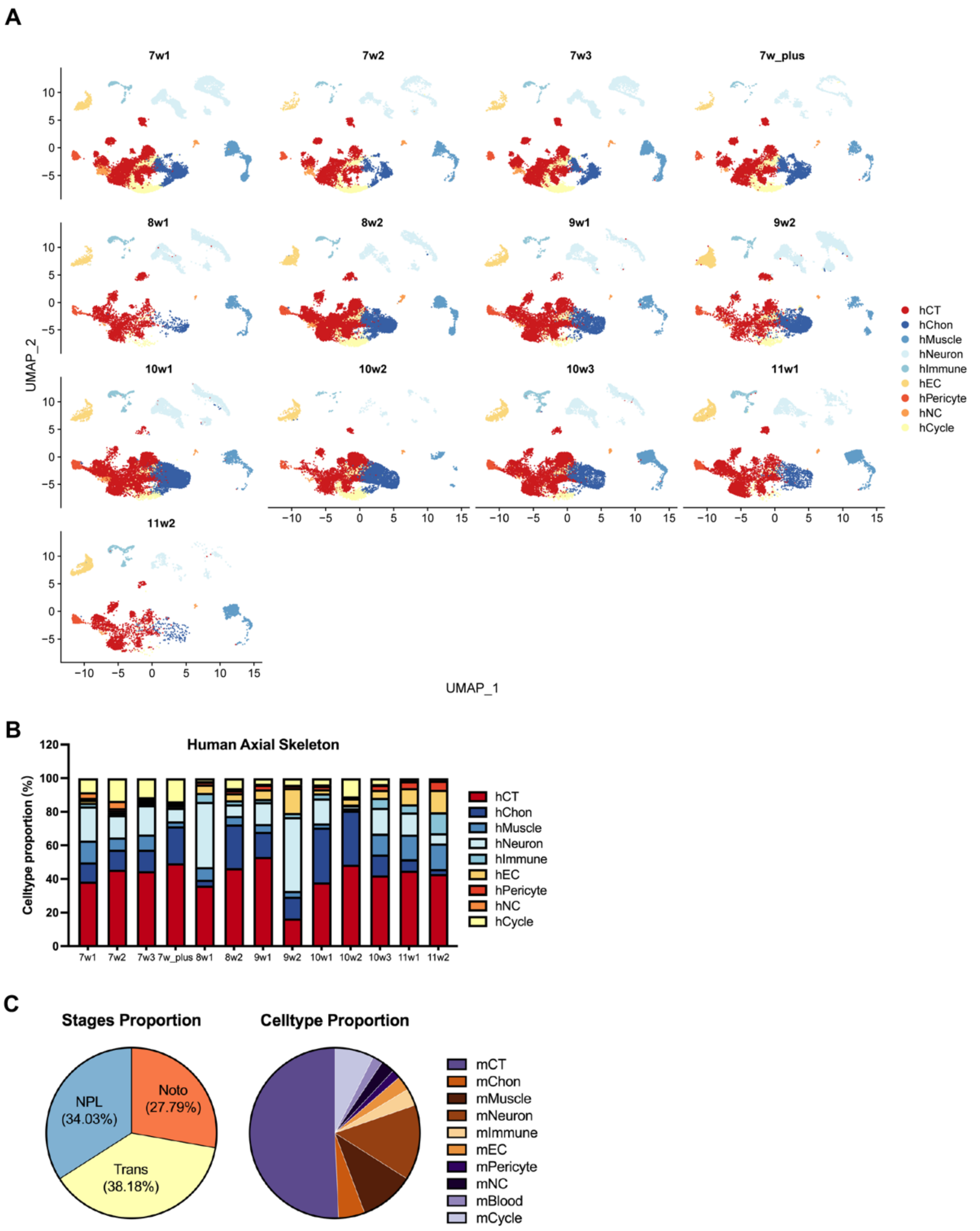

Figure S2. UMAPs (A), bar plot (B), and pie charts (C) showing the distribution of different clusters in each sample.

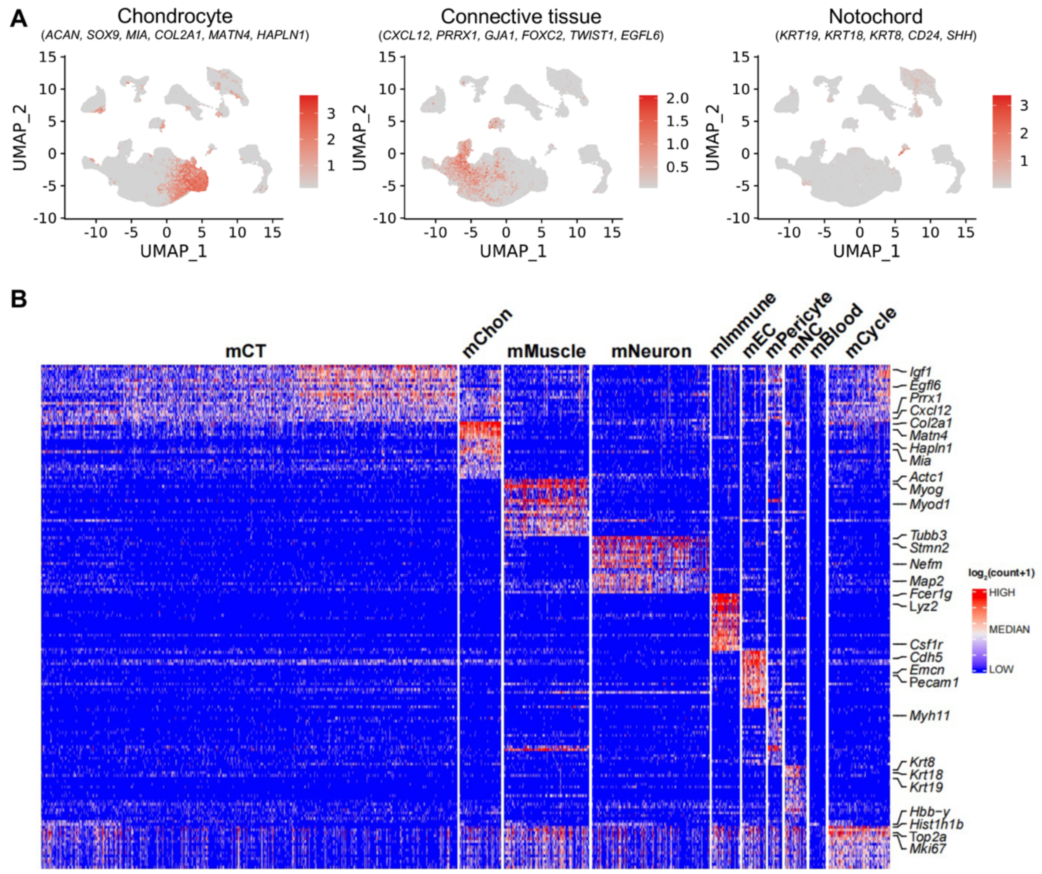

Figure S3. A) The average expression of curated feature genes for indicated cell clusters defined in **Figure 1C** on the UMAP. **B)** Heatmap showing the scaled expression of differentially expressed genes for each mouse cluster.

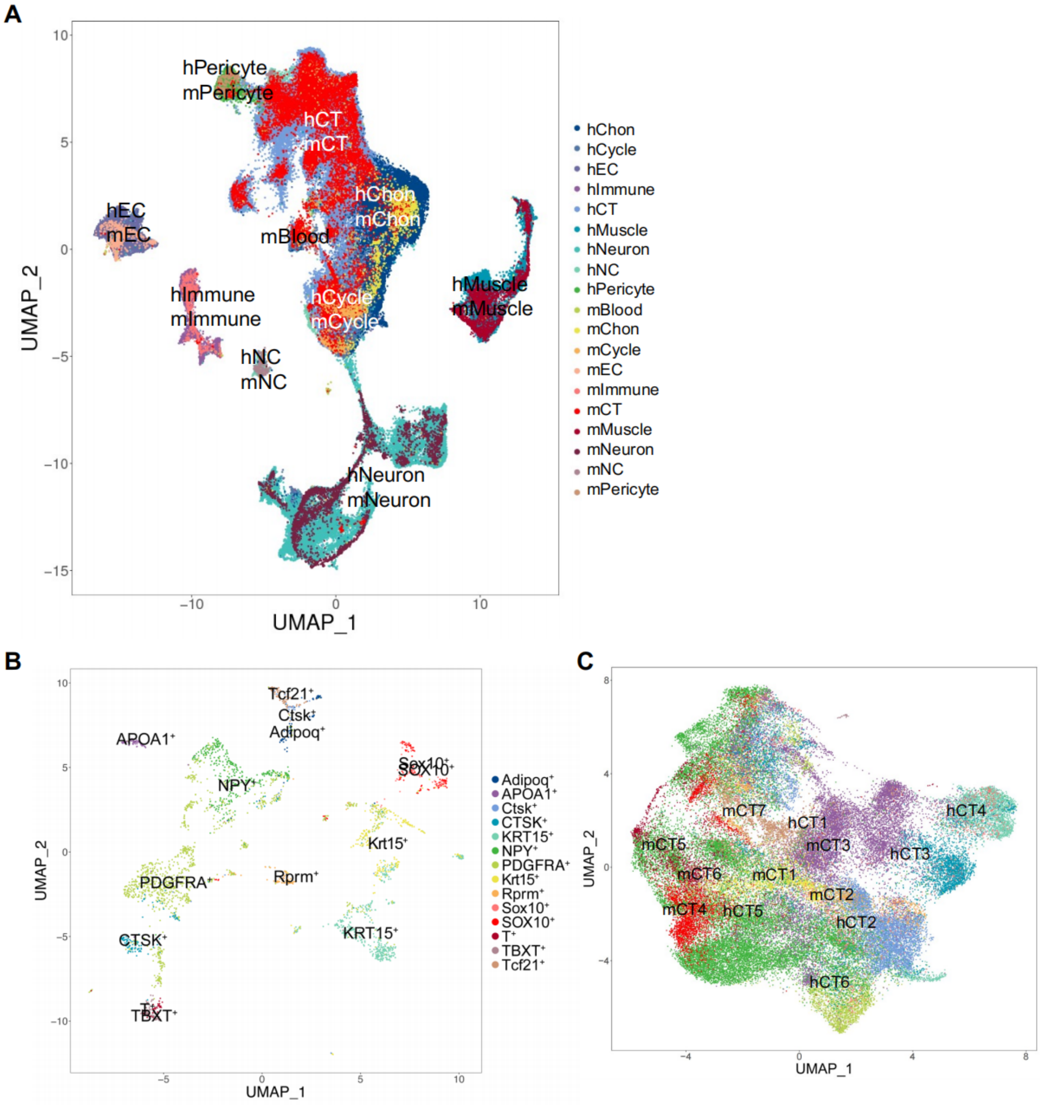

Figure S4. Integrated UMAP of human and mouse developing axial skeleton cells.

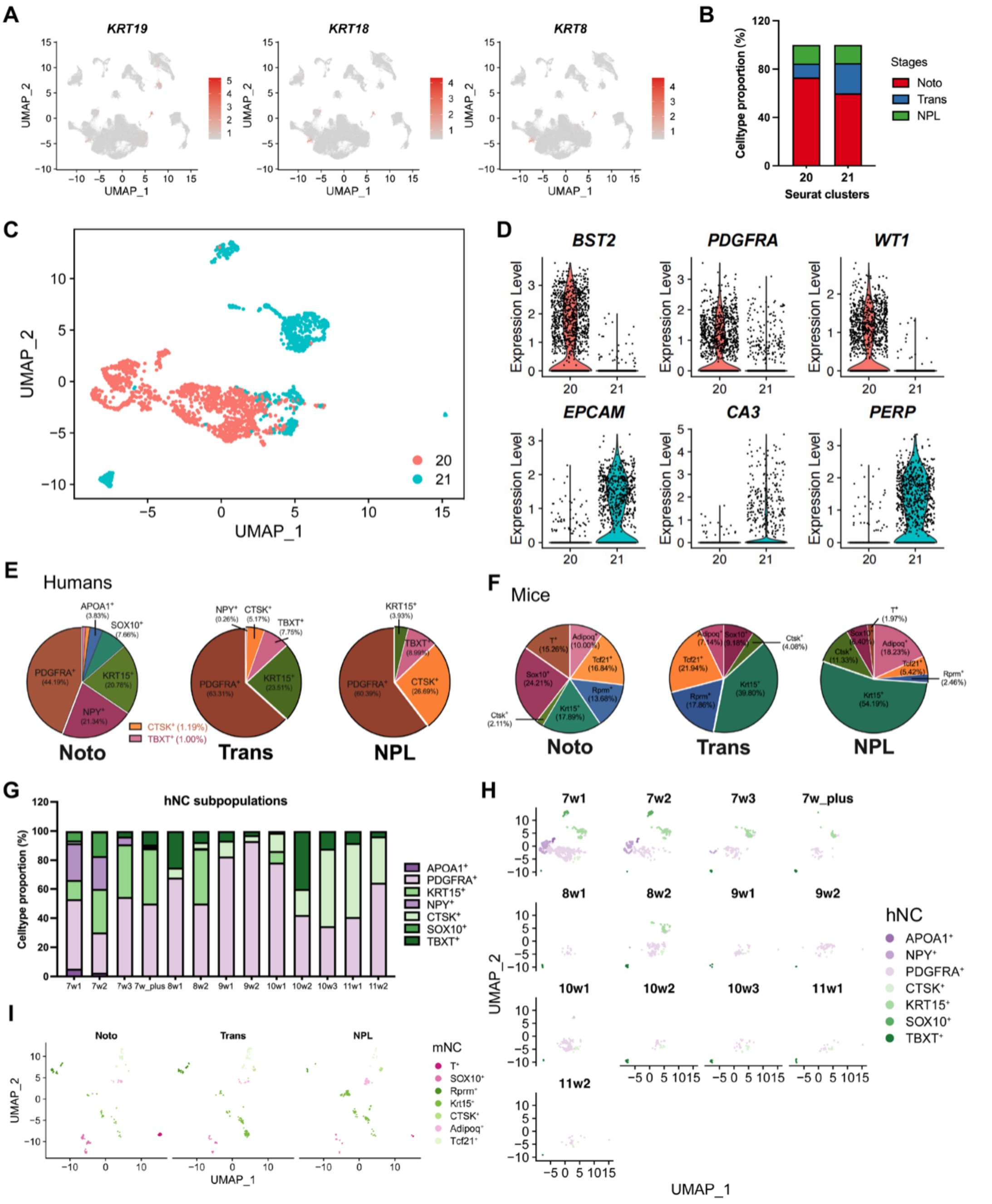

Figure S5. A) UMAPs showing the expressions of *KRT19*, *KRT18*, and *KRT8*. **B)** Bar plot showing the distribution of clusters 20 and 21 at the different developmental stages. **C)** UMAP showing the distribution of clusters 20 and 21. **D)** Violin plots showing the expressions of *BST2*, *PDGFRA*, *WT1*, *EPCAM*, *CA3*, and *PERP* in indicated human clusters. **E, F)** Pie charts showing the distribution of indicated human (**E**) and mouse (**F**) developing NC/NP cells at the Noto, Trans and NPL stage.

G) Bar plot showing the distribution of the seven hNC subclusters in each sample. **H, I)** UMAPs showing the distribution of hNC subclusters **(H)** and mNC subclusters **(I)** in each sample.

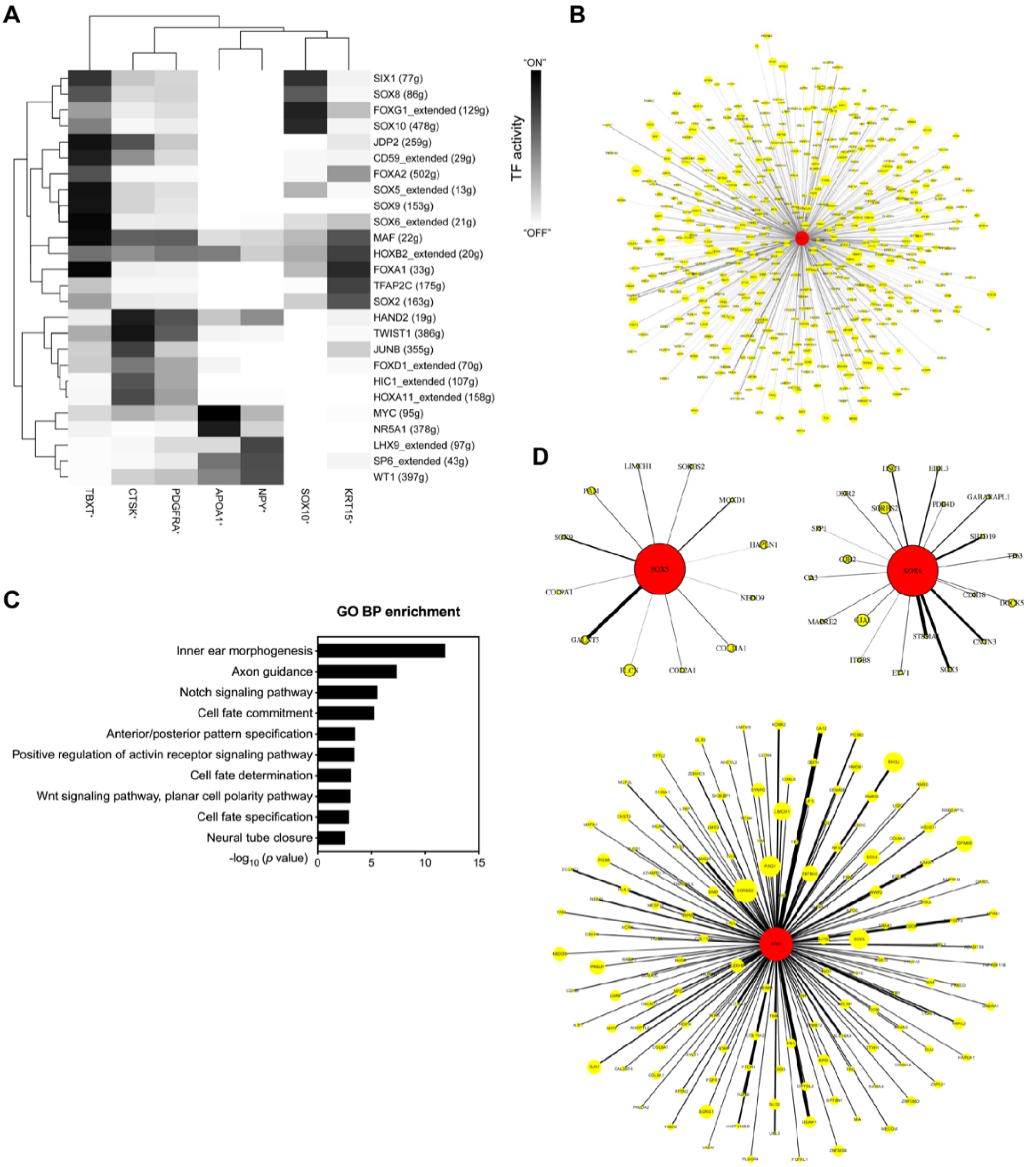

Figure S6. A) Heatmap revealing binary regulon activities analyzed with SCENIC in each hNC subclusters. "ON" indicates active regulons, and "OFF" indicates inactive regulons. **B)** The *SOX10* regulon networks in hNC subclusters. The TFs are in red, and the corresponding target genes are in yellow. **C)** Representation analysis of GO categories showing different functions for the target genes of the *SOX10* regulon. **D)** The *SOX5*, *SOX6*, *and SOX9* regulon networks in hNC subclusters. The TFs are in red, and the corresponding target genes are in yellow.

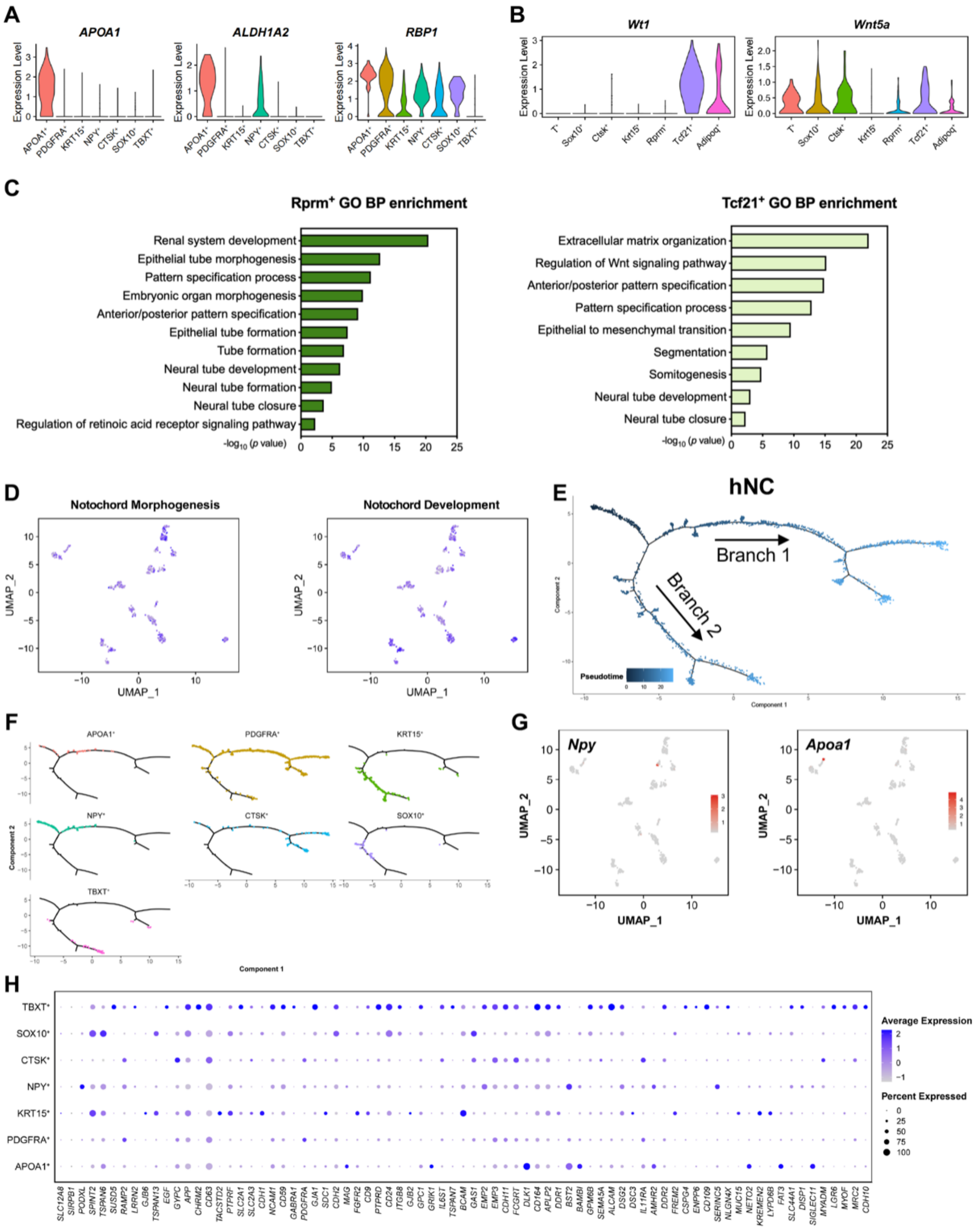

Figure S7. A) Violin plots showing the expressions of *APOA1*, *ALDH1A2*, and *RBP1* in the seven hNC subclusters. **B)** Violin plots showing the expressions of *Wt1* and *Wnt5a* in the seven mNC subclusters. **C)** Representation analysis of GO categories showing different functions for the Rprm$^+$ and Tcf21$^+$ subclusters. **D)** UMAP plot color-coded for pathway activities scored by GSVA per cell within mNC

subpopulations. **E, F)** Monocle pseudotime trajectory axis revealing the progression of the seven hNC subclusters. **G)** The expressions of *Npy* and *Apoa1* within mNC in the UMAP of Figure 2C. **H)** Dot plot showing the expressions of cell surface markers in the seven hNC subclusters.

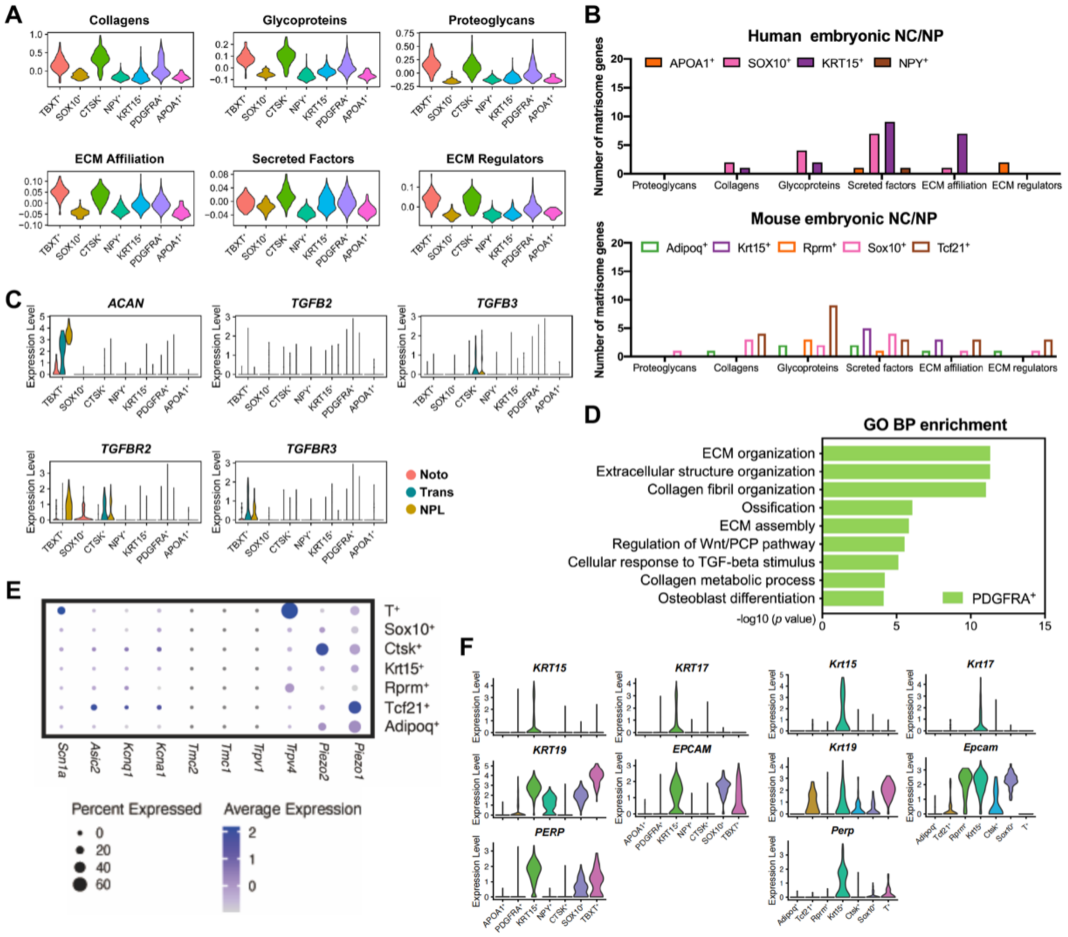

Figure S8. A) Violin plots showing the average expressions of six modules in the seven hNC subclusters. **B)** The number of expressed genes associated with six matrisome patterns in indicated human (upper) and mouse (lower) NC/NP cell subcluster. **C)** Violin plots showing the expressions of *ACAN*, *TGFB2*, *TGFB3*, *TGFBR2*, and *TGFBR3* among the seven hNC subpopulations. **D)** Representation analysis of GO categories showing different functions for the PDGFRA^{+} subcluster. **E)** Dot plot showing the mean expression of selected mechanosensitive ion channel genes among seven mouse NC/NP cell subpopulations. Dot size indicated the percentage of cells in subclusters with detected expression. **F)** Violin plots showing the expressions of *KRT15/Krt15*, *KRT17/Krt17*, *KRT19/Krt19*, *EPCAM/Epcam*, and

PERP/Perp among the human (left two panels) and mouse (right two panels) developing NC/NP cells.

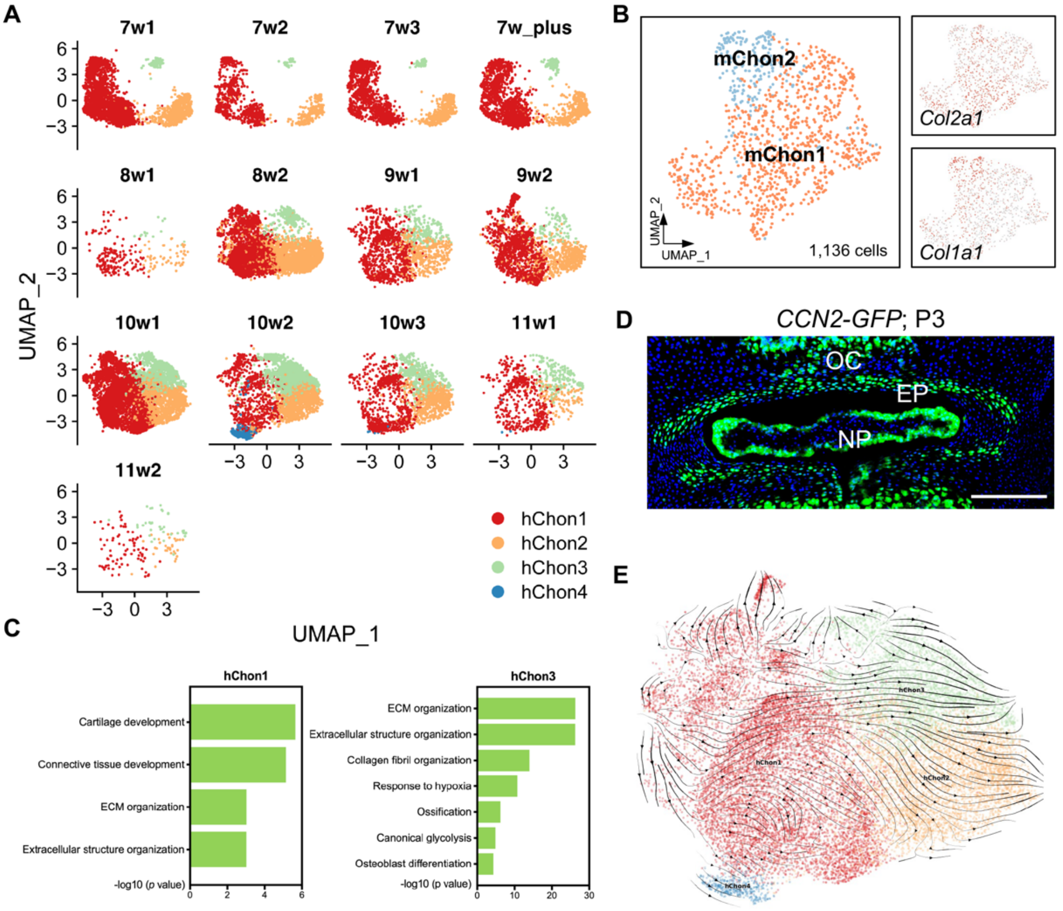

Figure S9. A) UMAPs showing the distribution of hChon subclusters in each sample. **B)** UMAP visualization of the two vertebral chondrocyte subclusters during mouse early IVD formation (left) and UMAP plots showing the expressions of *Col2a1* and *Col1a1* on the UMAP (right). **C)** Representation analysis of GO categories showing different functions for the hChon1 (left) and the hChon3 (right). **D)** Representative fluorescent images of the postnatal *Ccn2-GFP* mouse IVD section. EP, end plate; NP, nucleus pulposus; OC, ossification center. Scale bar, 200 μm. **E)** Visualization for dynamic velocities projected into the UMAP-based embedding.

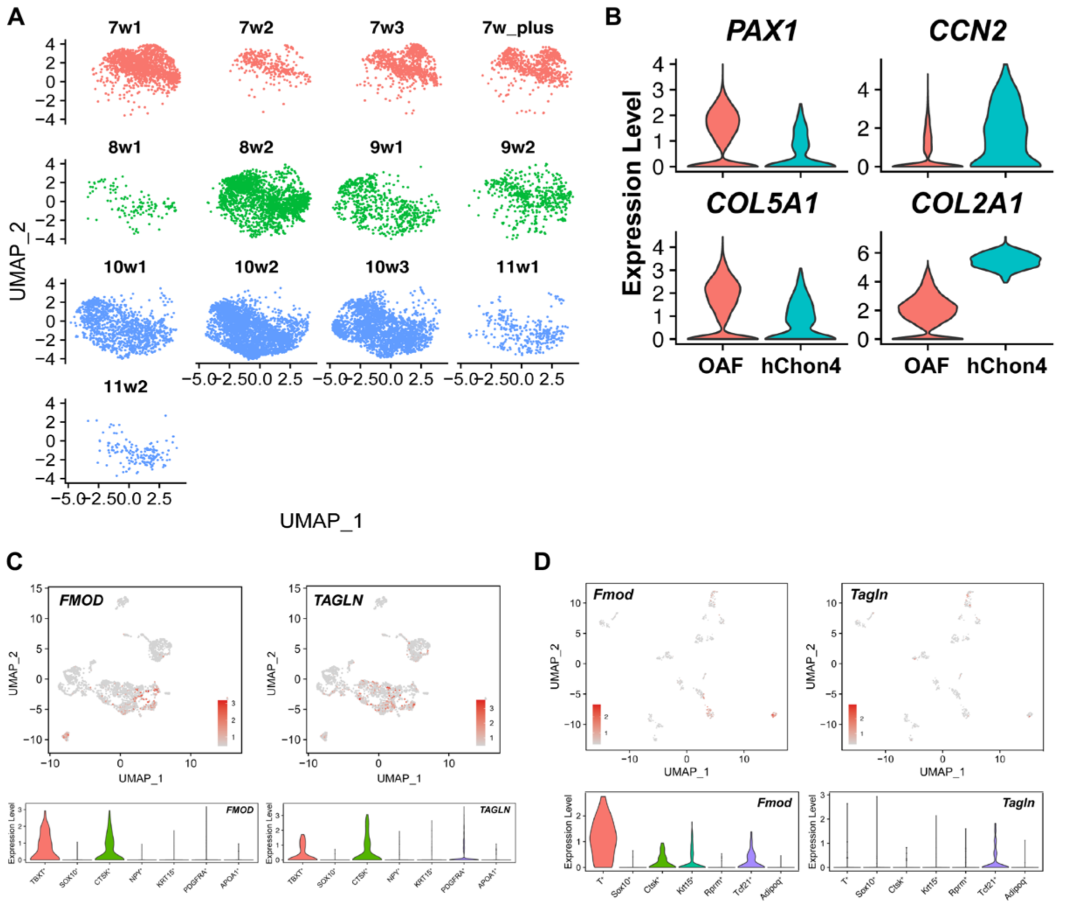

Figure S10. A) UMAPs showing the distribution of the hOAF in each sample colored by developmental stages. **B)** Violin plots showing the expressions of *PAX1*, *CCN2*, *COL5A1*, and *COL2A1* in the OAF and the hChon4 subpopulations. **C, D)** UMAPs (upper) and violin plots (lower) showing the expressions of *FMOD/Fmod* and *TAGLN/Tagln* in hNC (**C**) and mNC (**D**) subclusters.

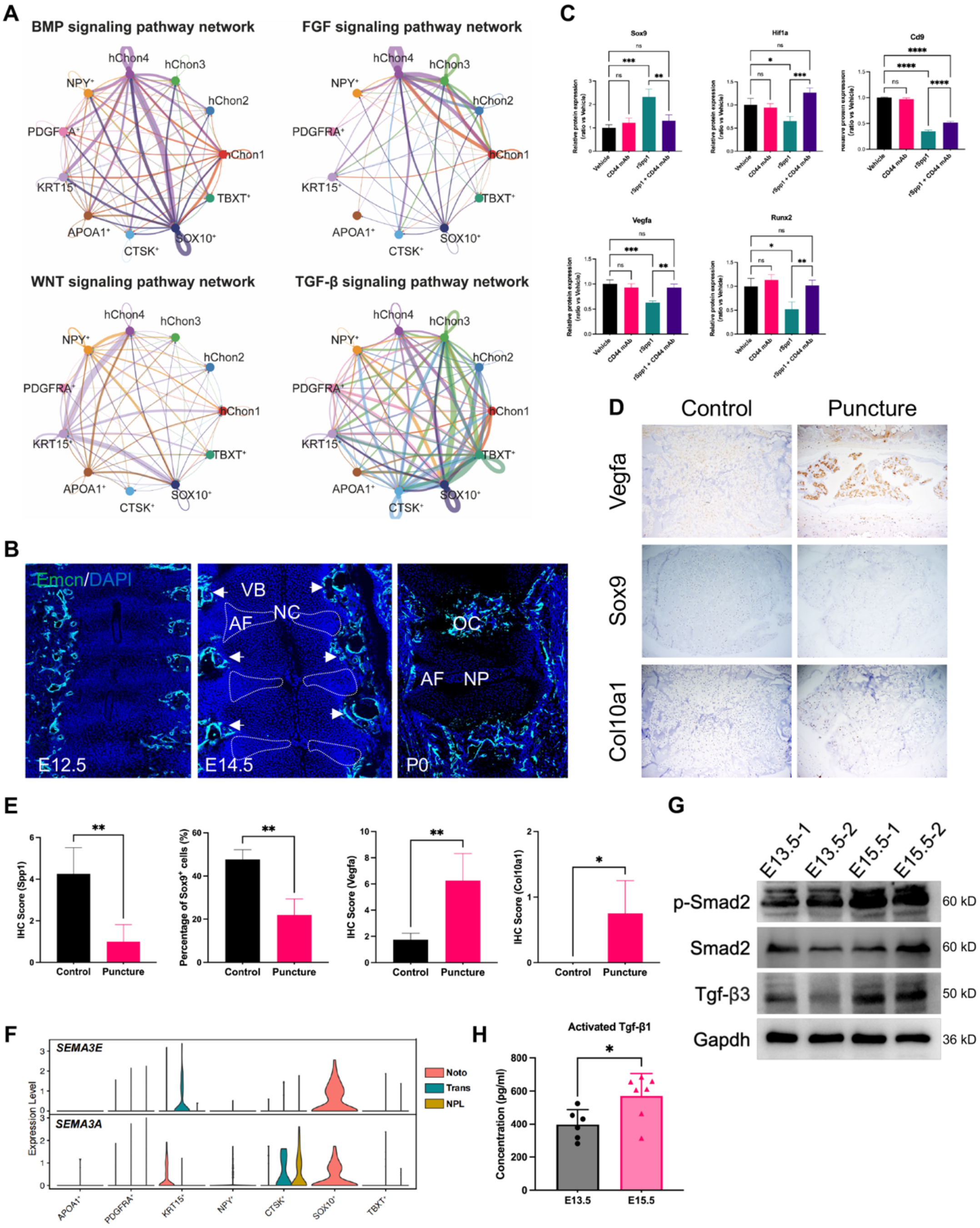

Figure S11. A) The indicated signaling pathway network between developing NC/NP and vertebral chondrocytes during human early IVD formation. Dots indicated cell subpopulations. The thickness of the directed line indicated the relative quantity of significant ligand-receptor pairs between any two pairs of subpopulations. **B)** Representative IF images of Emcn in mouse axial skeleton sections at indicated

developmental stages. The dotted line indicated the annulus fibrosus region. The arrows indicated the immunofluorescent signals of Emcn surrounding vertebral bodies. AF, annulus fibrosus; VB, vertebral body; NC, notochord; NP, nucleus pulposus; OC, ossification center. **C)** Quantification results of the western blotting results in Figure 6H. The results were presented as mean ± SD, n = 3, *p*-values were calculated using one-way ANOVA followed by Holm-Sidak test. **p*<0.05, ***p*<0.01, ****p*<0.001, *****p*<0.0001. **D)** Magnified IHC images of Vegfa, Sox9, and Col10a1 in rat IVDs. **E)** The IHC scores of Figure 6I and Figure S11D. The results were presented as mean ± SD, n = 3, *p*-values were calculated using the two-tailed independent Student's *t* test. **p*<0.05, ***p*<0.01, ****p*<0.001, *****p*<0.0001. **F)** Violin plots showing the expression of *SEMA3E* and *SEMA3A* among the defined human developing NC/NP subclusters. **G)** Western blotting results of Tgf-β3 and its downstream target (Smad2) in the mouse axial skeleton tissue homogenate at E13.5 and E15.5. **H)** The results of enzyme-linked immunosorbent assay (ELISA) of activated Tgf-β1 in the mouse axial skeleton tissue homogenate at E13.5 and E15.5. The results were presented as mean ± SD, n of E13.5 = 6, n of E15.5 = 7, *p*-values were calculated using the two-tailed independent Student's *t* test. **p*<0.05, ***p*<0.01, ****p*<0.001, *****p*<0.0001.

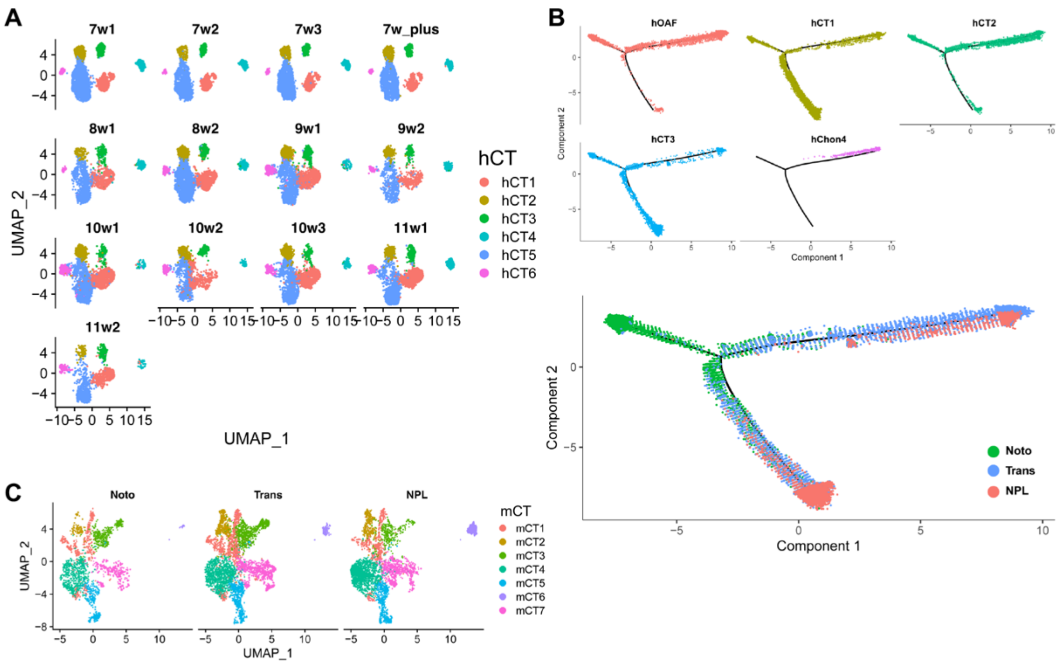

Figure S12. A) UMAPs showing the distribution of hCT subclusters in each sample. **B)** Monocle pseudotime trajectory axis revealing the progression among hCT1-3, hOAF, and hChon4. **C)** UMAPs showing the distribution of mCT subclusters in each sample.

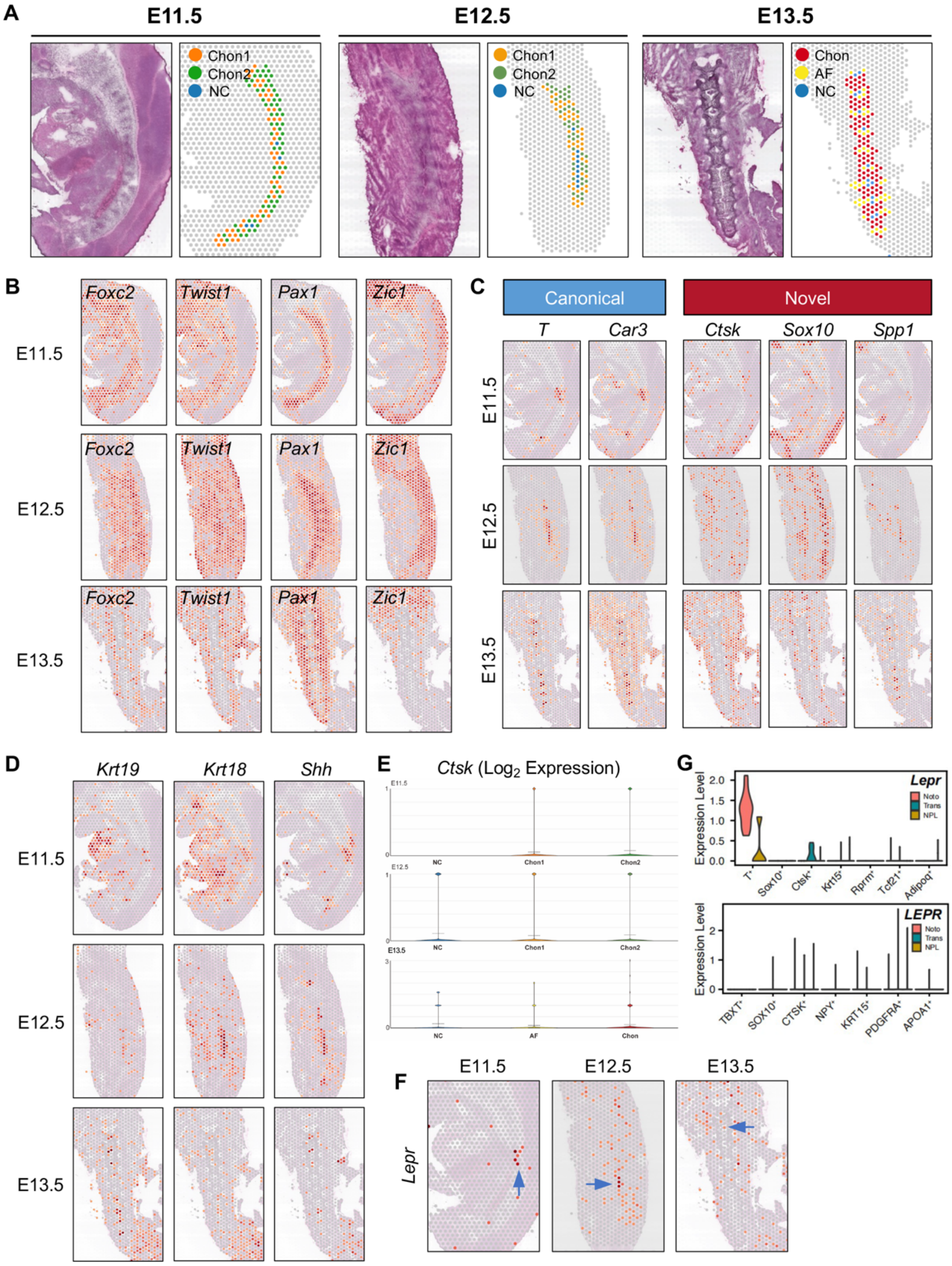

Figure S13. A) Projection of spots including defined mouse IVD cells on the histological section at the indicated developmental stages. **B)** Visualization of *Foxc2*, *Twist1*, *Pax1*, and *Zic1* expression on the histological sections at the indicated developmental stages. **C)** Visualization of *T*, *Car3*, *Ctsk*, *Sox10*, and *Spp1*

expressions in histological sections at the indicated developmental stages. **D)** Visualization of *Krt19, Krt18*, and *Shh* expression on the histological section at indicated developmental stages. **E)** Violin plots showing the expression of *Ctsk* among the spatially defined IVD cell clusters at indicated developmental stages. **F)** Visualization of *Lepr* expression on the histological section at indicated developmental stages. **G)** Violin plots showing the expression of *Lepr/LEPR* among the defined mouse (upper) and human (lower) developing NC/NP subclusters.

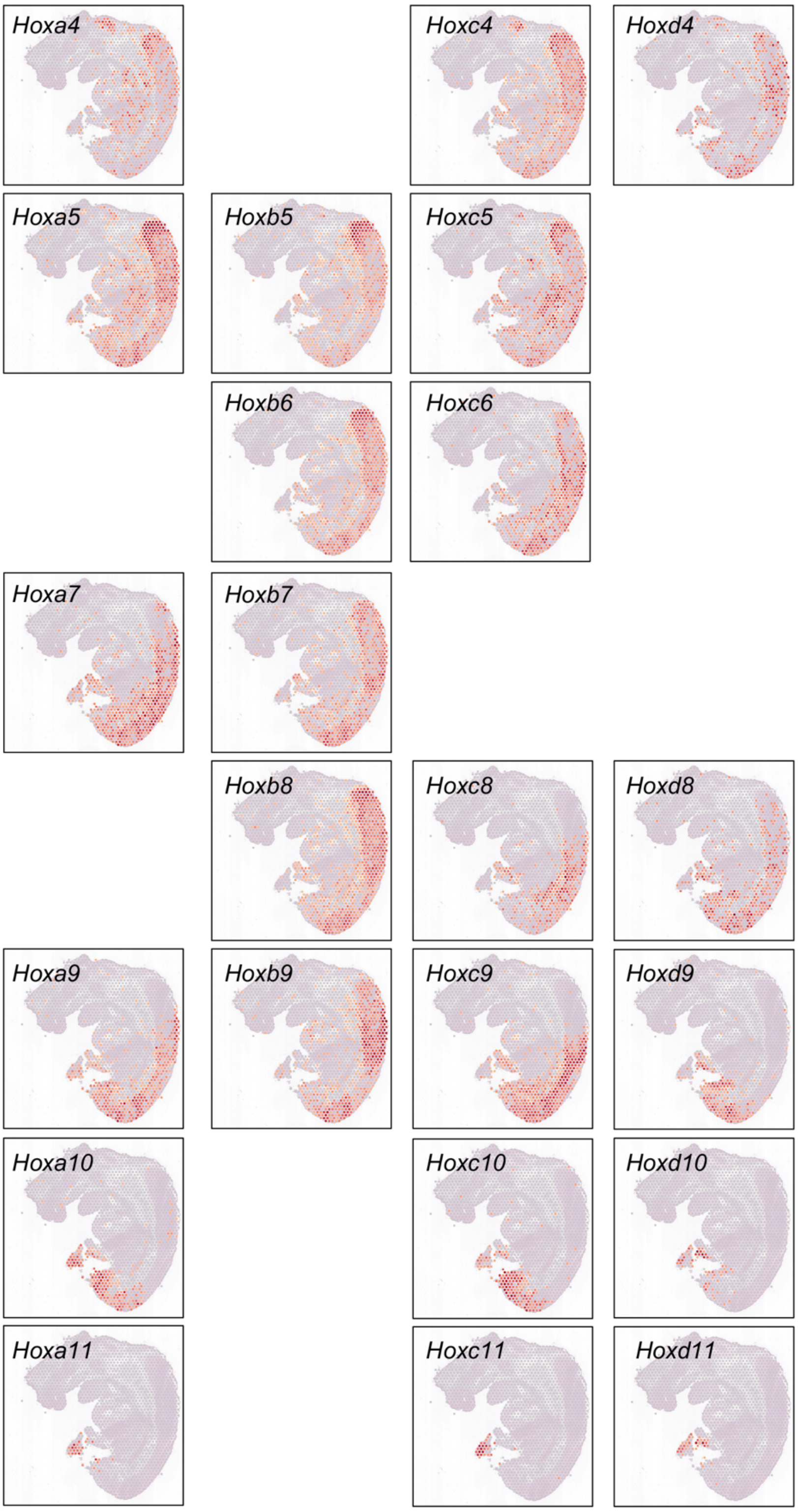

Figure S14. Visualization of *the Hox* family genes expression on the histological section at E11.5.

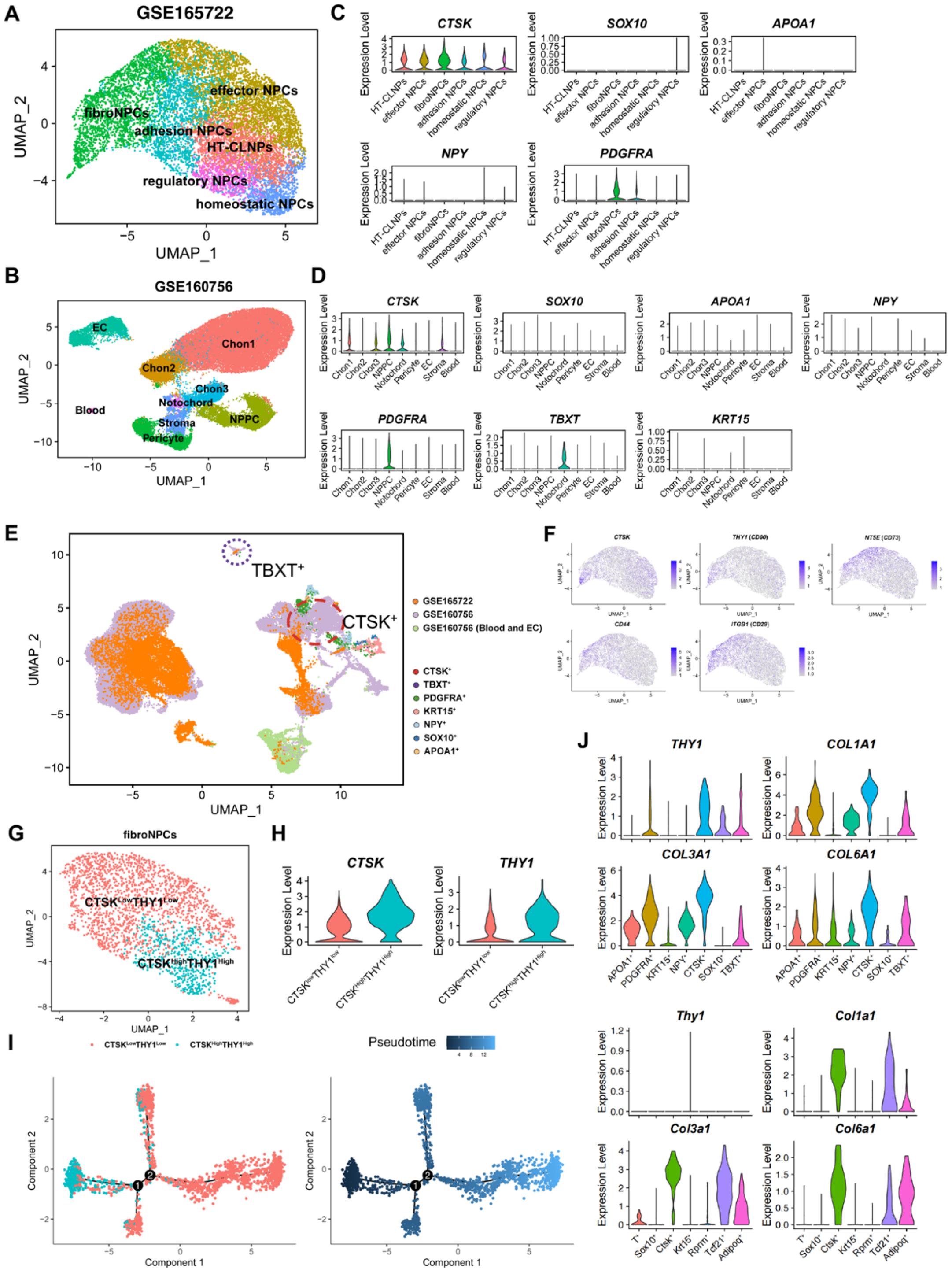

Figure S15. A, C) UMAPs of the two indicated IVD scRNA-seq datasets. **C, D)** Violin plots showing the expressions of the hNC signature genes in indicated datasets. **E)** The integrated UMAP of our hNC subpopulations and other two published IVD datasets. **F)** UMAPs showing the expressions of *CTSK* and the stem/progenitor

markers, such as *THY1 (CD90)*, *NT5E(CD73)*, *CD44*, and *ITGB1 (CD29)*. **G)** UMAP visualization of the CTSK^{High}THY1^{High} and the CTSK^{Low}THY1^{Low} clusters. **H)** Violin plots showing the expressions of *CTSK* and *THY1* in the CTSK^{High}THY1^{High} and the CTSK^{Low}THY1^{Low} clusters. **I)** Monocle pseudotime trajectory axis revealing the progression of the fibroNPCs. **J)** Violin plots showing the expressions of *THY1/Thy1, COL1A1/Col1a1, COL3A1/Col3a1*, and *COL6A1/Col6a1* among the defined human (upper) and mouse (lower) developing NC/NP subclusters.

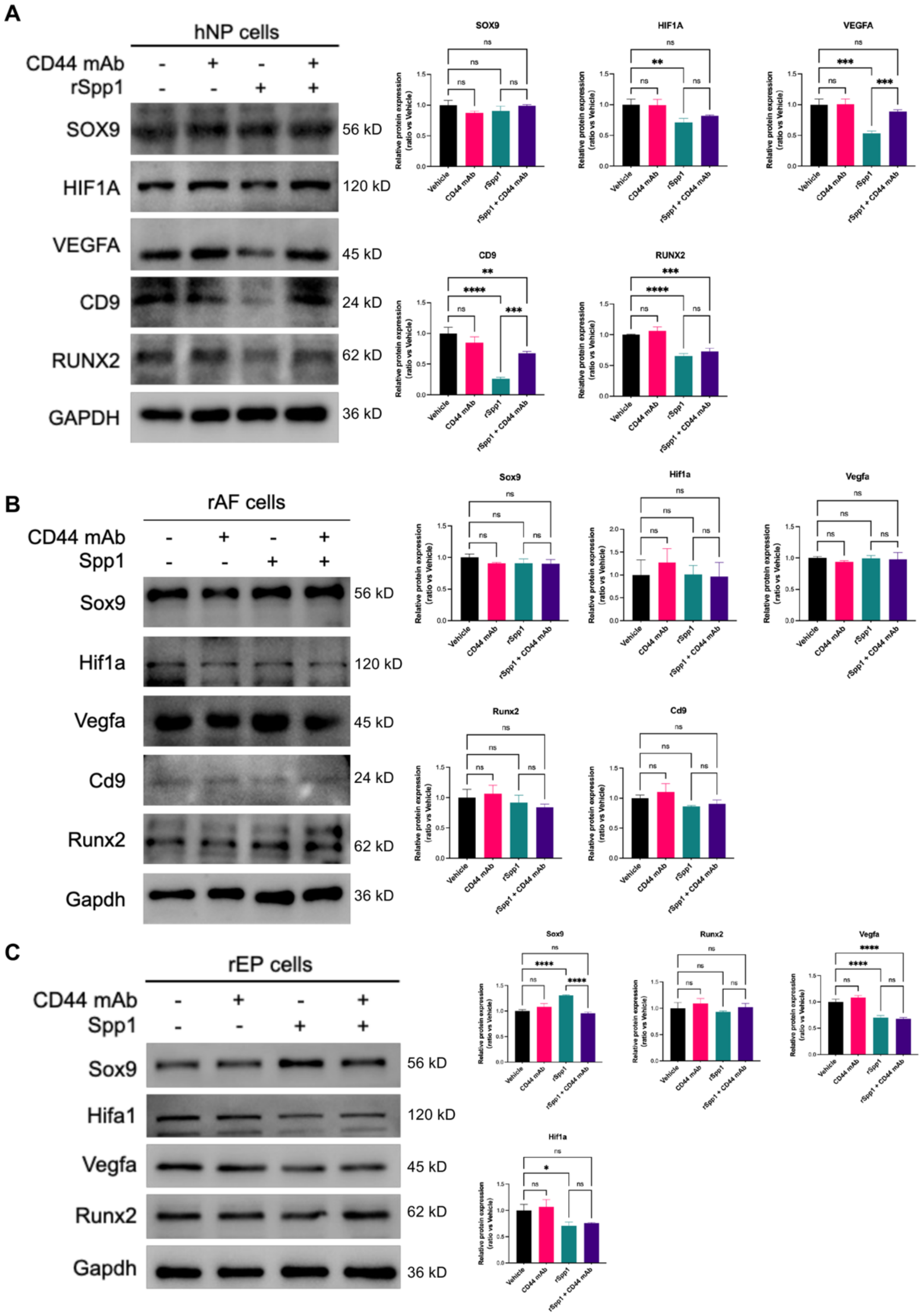

Figure S16. Western blotting results along with the quantification results. hNP, human nucleus pulposus; rAF, rat annulus fibrosus; rEP, rat end plate. The results were presented as mean ± SD, n = 3, *p*-values were calculated using one-way ANOVA followed by Holm-Sidak test. *$p<0.05$, **$p<0.01$, ***$p<0.001$, ****$p<0.0001$.

Table S1. DEGs of the defined human axial skeleton cell clusters.

Table S2. DEGs of the defined mouse axial skeleton cell clusters.

Table S3. DEGs of the seven human developing NC/NP cell subclusters.

Table S4. DEGs of the seven mouse developing NC/NP cell subclusters.

Table S5. Identified regulons along with their downstream targets in human NC/NP subclusters.

Table S6. The bulk RNA-seq FPKM of human developing NC/NP captured by LCM.

Table S7. DEGs of the four human vertebral chondrocyte subclusters.

Table S8. Scaled activity values of 75 regulons enriched in human vertebral chondrocyte subclusters.

Table S9. DEGs of the Seurat clusters in hCT (log$_2$FC >0.5, adjusted *P* value <0.01, and pct.1 >0.25).

Table S10. DEGs of the defined mesenchymal sclerotome subclusters (hCT1, hCT2 and hCT3; log$_2$FC >0.75, adjusted *P* value <0.01, and pct.1 >0.25).

Table S11. DEGs of the defined mCT subclusters.

Table S12. Locally distinguished features of the defined mouse IVD cell clusters at E11.5.

Table S13. Locally distinguished features of the defined mouse IVD cell clusters at E12.5.

Table S14. Locally distinguished features of the defined mouse IVD cell clusters at E13.5.
